# Multimodal mapping and analysis of the cyto- and receptorarchitecture of the human hippocampus

**DOI:** 10.1007/s00429-019-02022-4

**Published:** 2020-01-18

**Authors:** Nicola Palomero-Gallagher, Olga Kedo, Hartmut Mohlberg, Karl Zilles, Katrin Amunts

**Affiliations:** 1 Institute of Neuroscience and Medicine (INM-1), Research Centre Jülich, 52425 Jülich, Germany; 2 Department of Psychiatry, Psychotherapy and Psychosomatics, Medical Faculty, RWTH Aachen University, Aachen, Germany; 3 C. & O. Vogt Institute for Brain Research, Heinrich-Heine-University, 40225 Düsseldorf, Germany; 4 JARA-BRAIN, Jülich-Aachen Research Alliance, Jülich, Germany

**Keywords:** Hippocampus, Dentate gyrus, CA4, CA3, CA2, CA1, Prosubiculum, Subiculum, Presubiculum, Parasubiculum, Cytoarchitecture, Receptor autoradiography, Probabilistic maps

## Abstract

The human hippocampal formation is relevant for various aspects of memory and learning, and the different hippocampal regions are differentially affected by neuropsychiatric disorders. Therefore, the hippocampal formation has been subject of numerous cytoarchitectonic and other mapping studies, which resulted in divergent parcellation schemes. To understand the principles of hippocampal architecture, it is necessary to integrate different levels of hippocampal organisation, going beyond one modality. We here applied a multimodal mapping approach combining cyto- and multi-receptorarchitectonic analyses, and generated probabilistic maps in stereotaxic space of the identified regions. Cytoarchitecture in combination with the regional and laminar distribution of 15 neurotransmitter receptors visualized by in vitro receptor autoradiography were analysed in seven hemispheres from 6 unfixed shock frozen and serially sectioned brains. Cytoarchitectonic delineations for generation of probabilistic maps were carried out on histological sections from ten fixed, paraffin embedded and serially sectioned brains. Nine cyto- and receptorarchitectonically distinct regions were identified within the hippocampal formation (i.e., fascia dentata, cornu Ammonis (CA) regions 1–4, prosubiculum, subiculum proper, presubiculum and parasubiculum), as well as the hippocampal-amygdaloid transition area and the periallocortical transsubiculum. Subsequently generated probabilistic maps quantify intersubject variability in the size and extent of these cyto- and receptorarchitectonically distinct regions. The regions did not differ in their volume between the hemispheres and gender. Receptor mapping revealed additional subdivisions which could not be detected by cytoarchitectonic analysis alone. They correspond to parcellations previously found in immunohistochemical and connectivity studies. The multimodal approach enabled the definition of regions not consistently reported, e.g., CA4 region or prosubiculum. The ensuing detailed probabilistic maps of the hippocampal formation constitute the basis for future architectonically informed analyses of in vivo neuroimaging studies.

**Electronic supplementary material:**

The online version of this article (10.1007/s00429-019-02022-4) contains supplementary material, which is available to authorized users.

## Introduction

The hippocampal formation, which comprises the hippocampus *proper* and the subicular complex, plays a crucial role in the formation, organization, and retrieval of memories, and is involved in the control of mood, alertness and attention (e.g., Sweatt 2010, McDonald and Hong 2013). Furthermore, in humans different aspects of memory and learning have been associated with distinct regions of the hippocampus and/or subicular complex (Zeineh et al. 2003; Eldridge et al. 2005; Bakker et al. 2008; Suthana et al. 2009). Human autopsy and in vivo volumetric studies of the hippocampal formation have shown that hippocampal regions are differentially affected by neuropsychiatric disorders (Fukutani et al. 1995; Sousa et al. 1999; Sapolsky 2000; Rössler et al. 2002; Lim et al. 2012). However, the results of these studies are often inconsistent and controversial. For example, there are differences in the criteria applied for the definition of both the outer and inner hippocampal borders (Konrad et al. 2009). To overcome such problems, the Hippocampal Subfields Group was formed as an international initiative to develop a harmonized protocol for segmentation of the hippocampal formation on high-resolution MRI (Wisse et al. 2017). Verification of regional borders as seen in MRI by microstructural studies is crucial in this effort. Cytoarchitectonic probabilistic maps of the hippocampal formation in stereotaxic space are a tool to correlate MRI delineations (e.g., Wisse et al. 2012; Beaujoin et al. 2018) with cytoarchitecture. However, currently available maps provide separate delineations for the dentate gyrus (DG), the *cornu Ammonis* (CA) region and the subicular complex, but not subdivisions of the latter two (Amunts et al. 2005).

**Fig. 1:**
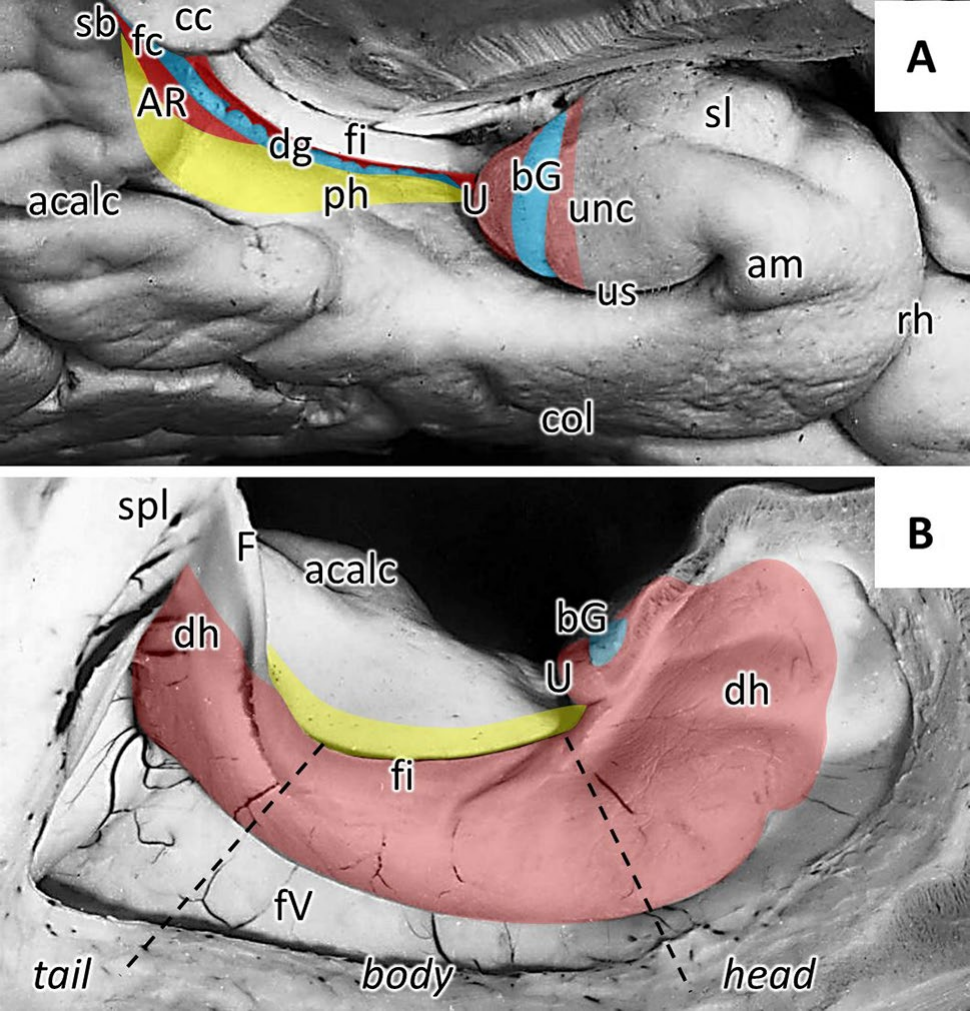
Hippocampal formation relative to macroanatomic landmarks. Medial (**a**) and intraventricular (**b**) views of the inferomedial portion of the temporal lobe. The location and extent of the dentate gyrus is highlighted in blue, that of the cornu Ammonis in red, and that of the subicular complex in yellow. *acalc* anterior calcarine sulcus, *am* ambient gyrus, *AR* gyri of Andreas Retzius, *bG* band of Giacomini, *cc* corpus callosum, *col* collateral sulcus, *dg* dentate gyrus, *dh* digitationes hippocampi, *F* fornix, *fi* fimbria, *fc* fasciola cinerea, *fV* floor of the lateral ventricle, *ph* parahippocampal gyrus, *rh* rhinal sulcus, *sb* subsplenial gyrus, *sl* semilunar gyrus, *spl* splenium of the corpus callosum, *U* uncal apex, *unc* uncinate gyrus, *us* uncinate sulcus

The macro- and microscopic organization of the hippocampal formation has been comprehensively described in multiple reviews and monographies (Duvernoy 2005; DeFelipe et al. 2007; Nieuwenhuys et al. 2008; Insausti and Amaral 2012). It is a convoluted gray matter structure encompassing three architectonically distinct regions: the fascia dentata (FD), the CA region (which can be subdivided into the CA1–CA4 fields), and the subicular complex. This cortical strip undergoes a complex in-folding process during embryogenesis which results in the FD and CA (together with the subicular complex) forming two interlocking C's in such a way that the FD is seen as the reversed C when viewing the hippocampal formation in coronal sections. The concavity formed by the FD envelops the CA4 segment of the CA region, and the combination of these two cytoarchitectonically distinct entities forms the macroscopically identifiable DG (Zilles et al. 2015). The walls of the hippocampal fissure, which separates the dentate gyrus from the CA1–CA3 fields and the subiculum, becomes partially fused during development. The hippocampal formation is located on the inferomedial aspect of the hemisphere (Fig. 1a), where it bulges into the temporal horn of the lateral ventricle and arches around the mesencephalon. It has been divided medio-laterally into intra- and extra-ventricular portions, and rostro-caudally into anterior (hippocampal head), intermediate (hippocampal body) and posterior (hippocampal tail) segments (Fig. 1b). The head is the most voluminous portion of the hippocampus, and its most rostro-medial part is often fused with the rostrally adjacent amygdala. The intraventricular aspect of the head is differentiated into the digitationes hippocampi, which are visibly macroscopically in the form of several lobules separated by small sulci (Fig. 1b). Its lateral aspect is associated with the caudal portion of the uncus, which results from the curling of the parahippocampal gyrus back onto itself (Fig. 1a). The uncal apex is occupied by the CA, and is delimited rostrally by the superficial segment of the FD, which follows a vertical route along the uncal surface and corresponds to the band of Giacomini (Fig. 1a). The hippocampal body is sagittally oriented, and its intraventricular part forms a smooth and strongly convex protrusion into the floor of the lateral ventricle. The extraventricular portion of the hippocampal body is reduced in size, being limited to the DG and fimbria (Fig. 1a). In humans, the narrow DG segment visible on the temporal lobe surface has a characteristic toothed appearance, and was thus named *margo denticulatus* (Klingler 1948), whereby the rounded protrusions which form the dentes of the DG decrease gradually in size when moving caudally. The hippocampal tail occupies the posterior part of the hippocampal arc and, as described above for the head, its intraventricular portion displays digitationes hippocampi (Fig. 1b). The hippocampal tail has been divided into initial, middle and terminal segments, with the first being found adjacent to the hippocampal body, and the last located ventral to the splenium of the corpus callosum. In the anterior segment the DG displays small dentes, but in the middle segment, the *margo denticulatus* becomes smooth and narrow, and forms the *fasciola cinerea*, i.e., segment of DG visible on the temporal lobe surface at the most posterior part of the hippocampal formation (Fig. 1a). In the hippocampal tail, CA1 appears progressively at the surface of the parahippocampal gyrus and sometimes produces rounded bulges, i.e., the gyri of Andreas Retzius (1896), which are separated from the *fasciola cinerea* by the superficial hippocampal sulcus (Fig. 1a). The terminal segment of the hippocampal tail covers the inferior surface of the splenium and constitutes the subsplenial gyrus.

**Fig. 2:**
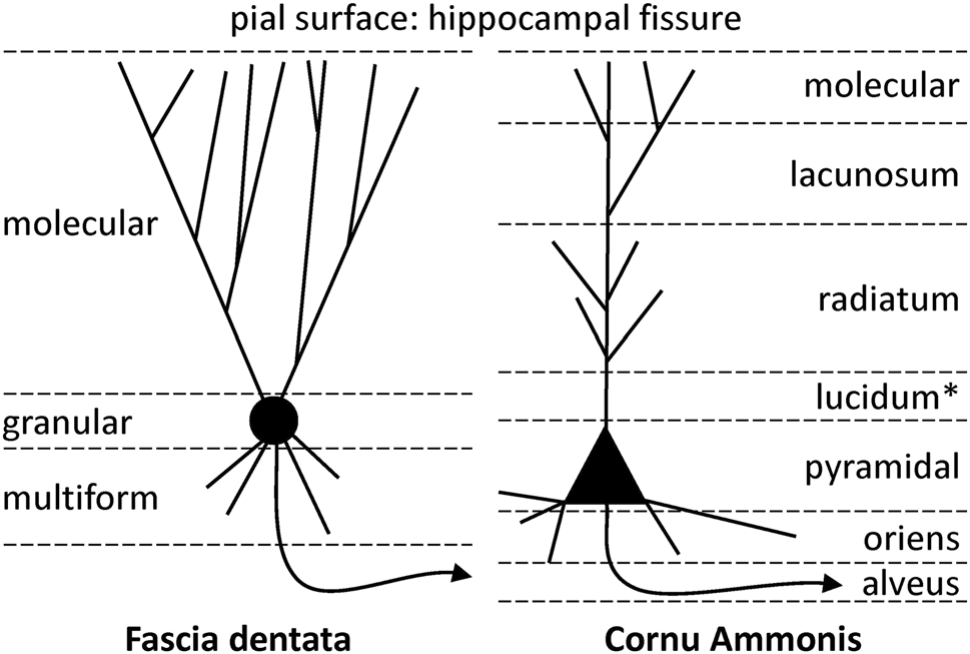
Schematic representation of the laminar structure of the fascia dentate and the cornu Ammonis. Note that the lucidum layer (highlighted by an asterisk) is only present in CA3, and the CA4 region only presents a single layer of modified pyramidal cells. Axon is indicated by curved line with an arrowhead

The FD and CA are archicortical in nature, whereas the subicular complex has been classified as periarchicortex (Zilles 2004). The FD displays the most rudimentary laminar structure of the hippocampal formation, with a superficial molecular layer followed by a very thin and densely packed granular layer and a thin multiform layer (Fig. 2) which abuts the CA4 region. Although six layers may be identified within CA (alveus, oriens, pyramidal, radiatum, lacunosum, and molecular; Fig. 2), they are often merged into three major layers: a broad pyramidal layer with the cell bodies of pyramidal neurons flanked by two molecular layers. The deeper molecular layer is composed of the oriens and alveus layers, and contains the basal dendrites and the axon of pyramidal cells. The superficial molecular layer encompasses the radiatum, lacunosum and molecular layers into which the apical dendrites of the pyramids extend. In the CA3 region an additional layer can be identified between the pyramidal and radiatum layers, i.e., the lucidum layer, which is targeted by the axons of the FD granular cells.

The hippocampal formation has been subject of numerous mapping studies which have resulted in divergent parcellation schemes. Lorente de Nó (1934) identified four regions within CA, i.e., CA1, CA2, CA3 and CA4. However, CA2 is not always accepted as a separate region, but has been merged with CA3 by some authors (von Economo and Koskinas 1925; Stephan 1975). CA4 is not universally accepted as a distinct region either, and merged with the multiform layer of FD to form the hilus of the DG (Vogt and Vogt 1919; von Economo and Koskinas 1925; Rose 1938; Amaral and Inausti 1990). Likewise, discrepancies also exist concerning the number of subregions which can be defined within a given CA region. For example, whereas Lorente de Nó (1934) identified three subfields within CA1, Vogt and Vogt (1919) described the existence of six subfields. The picture is similar when moving medially from the hippocampus into the subicular complex. Classical and contemporary architectonic studies brought forth different parcellation schemes, the most common of which includes the prosubiculum (ProS), subiculum proper (Sub), presubiculum (PreS) and parasubiculum (PaS). However, ProS and PaS are not always accepted as individual regions (Stephan 1975; Braak 1980; Amaral and Inausti 1990), and discrepancies also exist concerning the number of subregions that can be defined within a given region: e.g., two or three subdivisions within PreS (von Economo and Koskinas 1925; Rose 1927).

A multimodal strategy combining cyto- and receptorarchitectonic analyses has proven to be a powerful mapping tool to identify and characterize cortical areas as well as to reveal aspects concerning their functional hierarchical organization (Palomero-Gallagher and Zilles 2017; Zilles and Palomero-Gallagher 2017). Such an approach has not only enabled further confirmation of the location and extent of cytoarchitectonically identified cortical areas, but has also revealed further subdivisions within them (e.g., Geyer et al. 1996; Morosan et al. 2005; Palomero-Gallagher et al. 2008; Amunts et al. 2010). In a recent study of the human amygdala, such a multimodal approach enabled a better characterization of the paralaminar nucleus, which presents a molecular organization in-between that of nuclei belonging to the laterobasal and the superficial groups (Kedo et al. 2018).

The aim of the present study was twofold: (1) the multimodal definition and characterization of regions within the hippocampal formation, with particular attention as to whether further parcellations of the major hippocampal regions should be considered if multiple receptors are taken into consideration. (2) Application of novel workflows for the computation of probabilistic maps to create a revised and more detailed version of our previous map of the hippocampal formation (Amunts et al. 2005). Thus, whereas Amunts et al. (2005) provided delineations for the hippocampal-amygdaloid transition area (HATA), DG, CA and the subicular complex, but not for subdivisions of the latter two regions, the updated version resulting from the present study provides information concerning intersubject variability of the FD, CA4, CA3, CA2, CA1, ProS, Sub, PreS and PaS regions, and will enable future comparisons of cytoarchitectonically informed parcellation schemes in stereotaxic space with high resolution structural and/or functional imaging data.

## Materials and methods

### Tissue

We examined a total of 16 human post-mortem brains (Table 1) from subjects without a history of neurological or psychiatric disorders and obtained via body donors in accordance with the guidelines of the Ethics Committee of the University of Düsseldorf. Ten of these brains (5 males; mean age 64.9 ± 16.9 years; cases B01–B14 in Table 1) were processed for cytoarchitectonic analysis and subsequent computation of probabilistic 3D-maps. The remaining six brains (2 females; mean age: 76.3 ± 2.5 years; cases AR01–AR06 in Table 1) were processed for receptor autoradiographic analysis. In these latter cases, alternate sections were additionally processed for the visualization of cell bodies to compare the receptorarchitectonic findings with the cytoarchitecture of the hippocampal formation, i.e., the major criteria underlying the probability maps.

**Table 1:** Brains used for cyto- (brains B01–B14) and receptorarchitectonic (brains AR01–AR06) analysis of regions within the hippocampal formation

Case	Age (years)	Gender	Cause of death	Fresh weight (g)
B01	79	f	Bladder carcinoma	1350
B02	56	m	Rectal carcinoma	1270
B03	69	m	Vascular disease	1360
B04	75	m	Acute glomerulonephritis	1349
B05	59	f	Cardiorespiratory insufficiency	1142
B07	37	m	Cardiac arrest	1437
B08	72	f	Renal arrest	1216
B09	79	f	Cardiorespiratory insufficiency	1110
B13	39	m	Drowning	1234
B14	86	f	Cardiorespiratory insufficiency	1113
AR01	78	m	Multiorgan dysfunction	1326
AR02	75	f	Respiratory insufficiency	1280
AR03	79	m	Cardiac arrest	1477
AR04	77	m	Pulmonary oedema	1128
AR05	72	f	Melanoma	1326
AR06	77	m	Cardiac arrest	1272

Case numbering is according to designations of the brain bank from which the brains were selected. Only the right hemisphere of brains AR05 and AR06 were used in the present study

### Cytoarchitectonic analysis and probabilistic mapping

Brains were removed from the skull, fixed in formalin, scanned with a T1-weighted magnetic resonance sequence (3-D FLASH sequence covering the entire brain) before histological processing, then embedded in paraffin and serially sectioned (section thickness 20 µm) in the coronal plane with a large-scale microtome. Every 15th section was mounted on gelatine-coated slides and stained for cell bodies with a modified silver cell-body staining (Merker 1983). Every 60th section was mapped, and the borders of hippocampal regions CA1, CA2, CA3, CA4, FD, ProS, Sub, PreS and PaS were compared with the receptorarchitectonically defined borders (see below). The multimodally defined borders were finally traced on high resolution images of the histological sections of the ten formalin fixed brains using in-house software. These contours were used for the computation of volumes of the individual hippocampal areas using Cavalieri’s principle (Amunts et al. 2005) and for the 3D-reconstruction of mapped regions.

Whole brain volume representations were 3D-reconstructed by means of linear and non-linear registration algorithms using a modification of the workflow described in Amunts et al. (2005) using the images of the histological sections and the structural magnetic resonance (MR) data sets. In this improved approach, a new first step was introduced in which high resolution images of the sections were segmented into left and right hemispheres by means of labelled masks, and each hemisphere was iteratively corrected using a section-by-section elastic alignment of adjacent sections (Mohlberg et al. 2012). The ensuing histological volumes were then registered to the brain’s MR volume by linear and non-linear registration steps to eliminate distortions and shrinkage inevitably caused by histological techniques (Hömke 2006). Since each voxel in the histological volumes represented 20 µm, but resolution of the MR dataset was 1 mm, it was necessary to down-sample the histological volume, and, in contrast to the previously published maps of the hippocampal formation (Amunts et al. 2005), a continuous approach was chosen for this step. These down-sampled and morphologically rectified histological volumes together with the areal borders defined in each one of them (also at the 20 µm resolution level) were then spatially normalized to the T1-weighted single-subject template of the MNI (Collins et al. 1994) in anatomical MNI space (Amunts et al. 2005) using a nonlinear elastic registration algorithm (Henn et al. 1997; Hömke 2006). Corresponding areas of the different subjects could thus be superimposed in the anatomical MNI space to generate continuous probabilistic maps for each region. These maps describe for each voxel of the reference brain how many individual brains overlapped with their respective cytoarchitectonic region in that particular voxel. The extent of each region in the coronal, sagittal, and horizontal axis, as well as their centres of gravity were calculated in anatomical MNI coordinates for each of the examined brains individually. Finally, maximum probability maps were generated for each region by calculating for each voxel of the reference brain the highest probability to contain a given region (Eickhoff et al. 2005). The maximum probability maps thus provide a unique estimate of the extent and location of each region.

### Receptor autoradiography

Brains were bisected at autopsy, hemispheres cut into 2–3 cm thick slabs, frozen in isopentane at − 40 °C and serially sectioned in the coronal plane into 20 μm thick sections by means of a large-scale cryostat microtome. Alternating sections were processed for visualization of 15 different neurotransmitter receptor binding sites by means of quantitative in vitro receptor autoradiography according to previously published standard protocols (Zilles et al. 2002; Palomero-Gallagher and Zilles 2018); Table 2), or for the visualization of cell bodies (Merker 1983). In short, a binding protocol consists of three steps: (1) a pre-incubation to re-hydrate sections and remove endogenous ligands, (2) a main incubation to label binding sites with a tritiated ligand in the presence (non-specific binding) or absence (total binding) of an appropriate non-labelled displacer, and (3) a final rinsing step to stop binding and eliminate surplus radioactive ligands. Since non-specific binding was less than 5% of total binding in all cases, we considered the estimates of the total binding to be equal to specific binding.

**Table 2:** Protocols for receptor autoradiography. Substances listed between square brackets were only included in the buffer solution during the main incubation

Transmitter	Receptor	Ligand	Displacer	Incubation buffer	Preincubation	Main incubation	Final rinsing
Glutamate	AMPA	[^3^H] AMPA (10 nM)	Quisqualat (10 µM)	50 mM Tris–acetate (pH 7.2) [+ 100 mM KSCN]	3 × 10 min, 4 °C	45 min, 4 °C	(1) 4 × 4 sec, 4 °C (2) Acetone-glutaraldehyde (100 ml + 2.5 ml) 2 × 2 s, 22 °C
	Kainate	[^3^H] kainite (9.4 nM)	SYM 2081 (100 µM)	50 mM Tris–acetate (pH 7.1) [+ 10 mM Ca-acetate]	3 × 10 min, 4 °C	45 min, 4 °C	(1) 3 × 4 s, 4 °C (2) Acetone-glutaraldehyde (100 ml + 2.5 ml), 2 × 2 s, 22 °C
	NMDA	[^3^H] MK-801 (3.3 nM)	( +)MK-801 (100 µM)	50 mM Tris–acetate (pH 7.2) + 50 µM glutamate [+ 30 µM glycine + 50 µM spermidine]	15 min, 4 °C	60 min, 22 °C	(1) 2 × 5 min, 4 °C (2) Dip in distilled water, 22 °C
GABA	GABA_A_	[^3^H] Muscimol (7.7 nM)	GABA (10 µM)	50 mM Tris–citrate (pH 7.0)	3 × 5 min, 4 °C	40 min, 4 °C	(1) 3 × 3 s, 4 °C (2) Dip in distilled water, 22 °C
	GABA_B_	[^3^H] CGP 54626 (2 nM)	CGP 55845 (100 µM)	50 mM Tris–HCl (pH 7.2) + 2.5 mM CaCl_2_	3 × 5 min, 4 °C	60 min, 4 °C	(1) 3 × 2 s, 4 °C (2) Dip in distilled water, 22 °C
	GABA_A_/BZ	[^3^H] Flumazenil (1 nM)	Clonazepam (2 µM)	170 mM Tris–HCl (pH 7.4)	15 min, 4 °C	60 min, 4 °C	(1) 2 × 1 min, 4 °C (2) Dip in distilled water, 22 °C
Acetylcholine	M_1_	[^3^H] Pirenzepine (1 nM)	Pirenzepine (2 µM)	Modified Kreb’s buffer (pH 7.4)	15 min, 4 °C	60 min, 4 °C	(1) 2 × 1 min, 4 °C (2) Dip in distilled water, 22 °C
	M_2_	[^3^H] Oxotremorine-M (1.7 nM)	Carbachol (10 µM)	20 mM HEPES-Tris (pH 7.5) + 10 mM MgCl_2_ + 300 nM pirenzepine	20 min, 22 °C	60 min, 22 °C	(1) 2 × 2 min, 4 °C (2) Dip in distilled water, 22 °C
	M_3_	[^3^H] 4-DAMP (1 nM)	Atropine sulfate (10 µM)	50 mM Tris–HCl (pH 7.4) + 0.1 mM PSMF + 1 mM EDTA	15 min, 22 °C	45 min, 22 °C	(1) 2 × 5 min, 4 °C (2) Dip in distilled water, 22 °C
	Nic α_4_/β_2_	[^3^H] Epibatidine (0.5 nM)	Nicotine (100 µM)	15 mM HEPES (pH 7.5) + 120 mM NaCl + 5.4 mM KCl + 0.8 mM MgCl_2_ + 1.8 mM CaCl_2_	20 min, 22 °C	90 min, 22 °C	(1) 5 min, 4 °C (2) Dip in distilled water, 22 °C
Serotonin	5-HT_1A_	[^3^H] 8-OH-DPAT (1 nM)	5-Hydroxy-tryptamine (1 µM)	170 mM Tris–HCl (pH 7.4) [+ 4 mM CaCl_2_ + 0.01% ascorbate]	30 min, 22 °C	60 min, 22 °C	(1) 5 min, 4 °C (2) Dip in distilled water, 22 °C
	5-HT_2_	[^3^H] Ketanserine (1.14 nM)	Mianserin (10 µM)	170 mM Tris–HCl (pH 7.7)	30 min, 22 °C	120 min, 22 °C	(1) 2 × 10 min, 4 °C (2) Dip in distilled water, 22 °C
Norepinephrine	α_1_	[^3^H] Prazosin (0.2 nM)	Phentolamine mesylate (10 µM)	50 mM Na/K-phosphate buffer (pH 7.4)	15 min, 22 °C	60 min, 22 °C	(1) 2 × 5 min, 4 °C (2) Dip in distilled water, 22 °C
	α_2_	[^3^H] RX 821002 (1.4 nM)	Phentolamine mesylate (10 µM)	50 mM Tris–HCl (pH 7.7) + 100 µM MnCl_2_	15 min, 22 °C	90 min, 22 °C	(1) 5 min, 4 °C (2) Dip in distilled water, 22 °C
Dopamine	D_1_	[^3^H] SCH 23390 (1.67 nM)	SKF 83566 (1 µM)	50 mM Tris–HCl (pH 7.4) + 120 mM NaCl + 5 mM KCl + 2 mM CaCl_2_ + 1 mM MgCl_2_	20 min, 22 °C	90 min, 22 °C	(1) 2 × 20 min, 4 °C (2) Dip in distilled water, 22 °C

*GABA*_*A*_*/BZ* GABA_A_ associated benzodiazepine binding sites

Radioactively labelled sections were co-exposed with plastic [^3^H]-standards (Microscales; Amersham, Braunschweig, Germany) of known radioactivity concentrations against tritium-sensitive films for 8–15 weeks. The ensuing autoradiographs were digitized and processed densitometrically (Zilles et al. 2002; Palomero-Gallagher and Zilles 2018). The plastic standards were used to compute a transformation curve indicating the relationship between grey values in the autoradiograph and receptor densities (fmol/mg protein) in the tissue. Images were subsequently linearized, contrast enhanced, smoothed, and pseudo-colour coded in a spectral order to optimize visualization of regional and laminar receptor distribution patterns.

### Multimodal border definition

Borders were first identified according to cytoarchitectonic criteria based on previous cyto- and pigment-architectonic studies in the hippocampus (von Economo and Koskinas 1925; Lorente de Nó 1934; Rose 1927, 1938; Braak 1978; Rosene and Van Hoesen 1987; Duvernoy 1988; Amaral and Inausti 1990; Amunts et al. 2005) and subicular complex (von Economo and Koskinas 1925; Stephan 1975; Braak 1980; Amaral and Inausti 1990), then compared with those revealed by differences in receptor distribution patterns. Cytoarchitectonically detected borders were mirrored by changes in the expression levels of multiple receptors. Furthermore, differences in receptor densities also enabled the subdivision of cytoarchitectonically defined regions.

Since cytoarchitectonic probabilistic mapping requires analysis of formalin-fixed brain tissue, while the use of deep frozen tissue is the prerequisite for quantitative in vitro receptor autoradiography, it is technically not possible to use the same brains for probabilistic cytoarchitectonic mapping and receptorarchitectonic studies.

### Statistical analyses

The volumes of each hippocampal region were analysed with respect to interhemispheric and gender differences using Monte-Carlo permutation tests. Prior to this analysis, all areal volumes were expressed as a fraction of total brain volume for each brain to adjust for differences in total brain size.

For the analysis of gender differences, we first computed the difference in the mean volumes between male and female subjects. Under the null-hypothesis of gender exchangeability, we then randomly reassigned each subject to one of the two groups (male/female) and re-computed the respective difference between the mean volumes of the ensuing randomly assembled groups. This difference obtained under the null-hypothesis that subjects' assignment to a gender group was recorded, and the procedure repeated 10^6^ times. The true gender difference was then considered significant if it was larger than 95% of the values under random (i.e., null hypothesis) distribution (*p* < 0.05; Bonferroni corrected for multiple comparisons).

In contrast to this between-subject design used to assess gender differences, the analysis of inter-hemispheric differences used a within-subject design. In particular, we first computed the difference between left and right regional volume for each subject. The mean of these values represents the average inter-hemispheric difference in the ensuing paired-test design. Under the null-hypothesis that there is no difference between the hemispheres and that side-labels may hence be freely exchangeable, we then randomly and independently across subjects designated the two measurements as "left" or "right". Again, the difference between "left" and "right" areal volume was computed for each subject, thus providing a difference value under the null-hypothesis that left and right values were not systematically different. This procedure was repeated 10^6^ times and true inter-hemispheric differences were then considered significant if it was larger than 95% of the values under random (i.e., null hypothesis) distribution (*p* < 0.05; Bonferroni corrected for multiple comparisons).

## Results

### Multimodal characterization of regions within the hippocampal formation

**Fig. 3:**
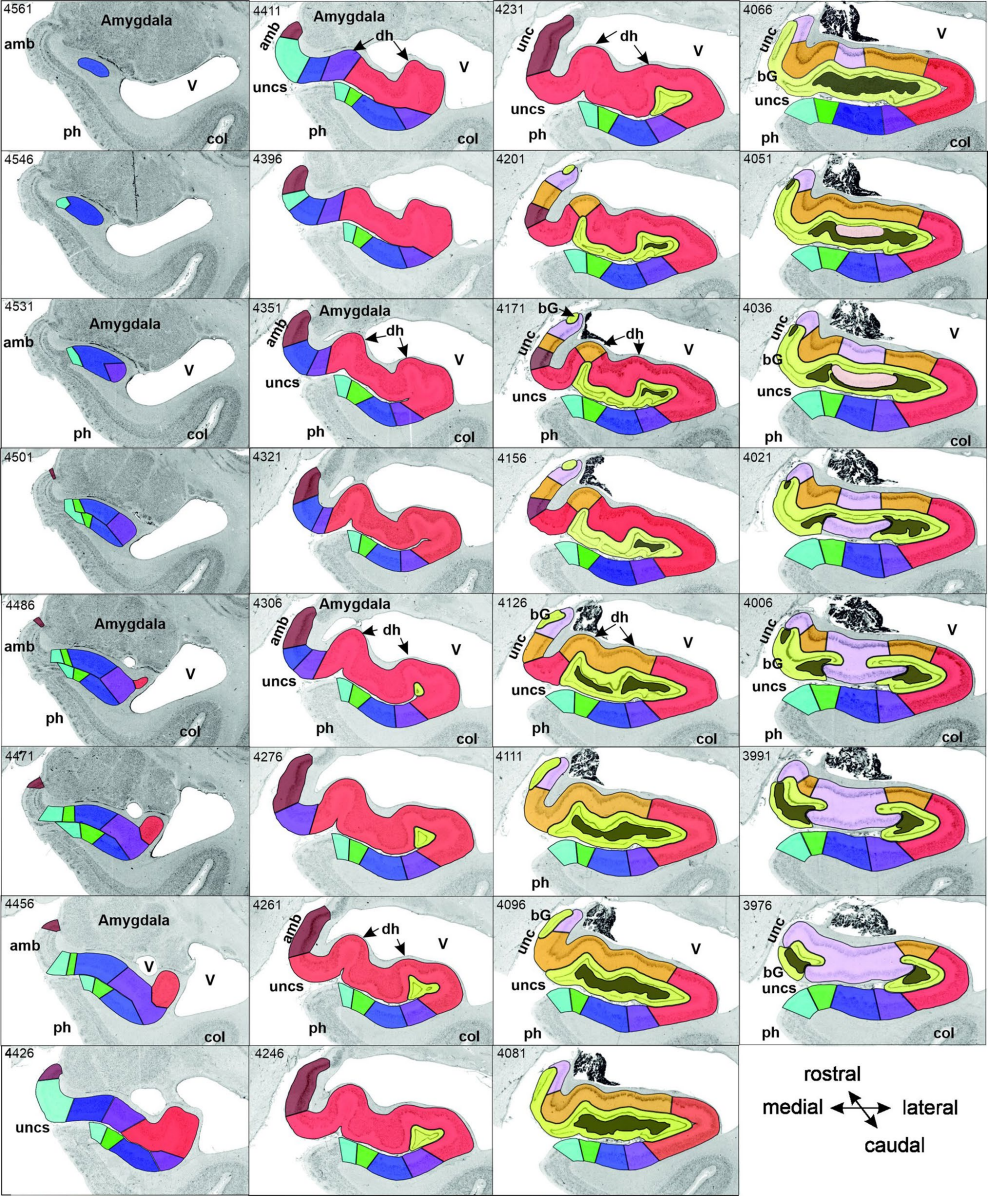

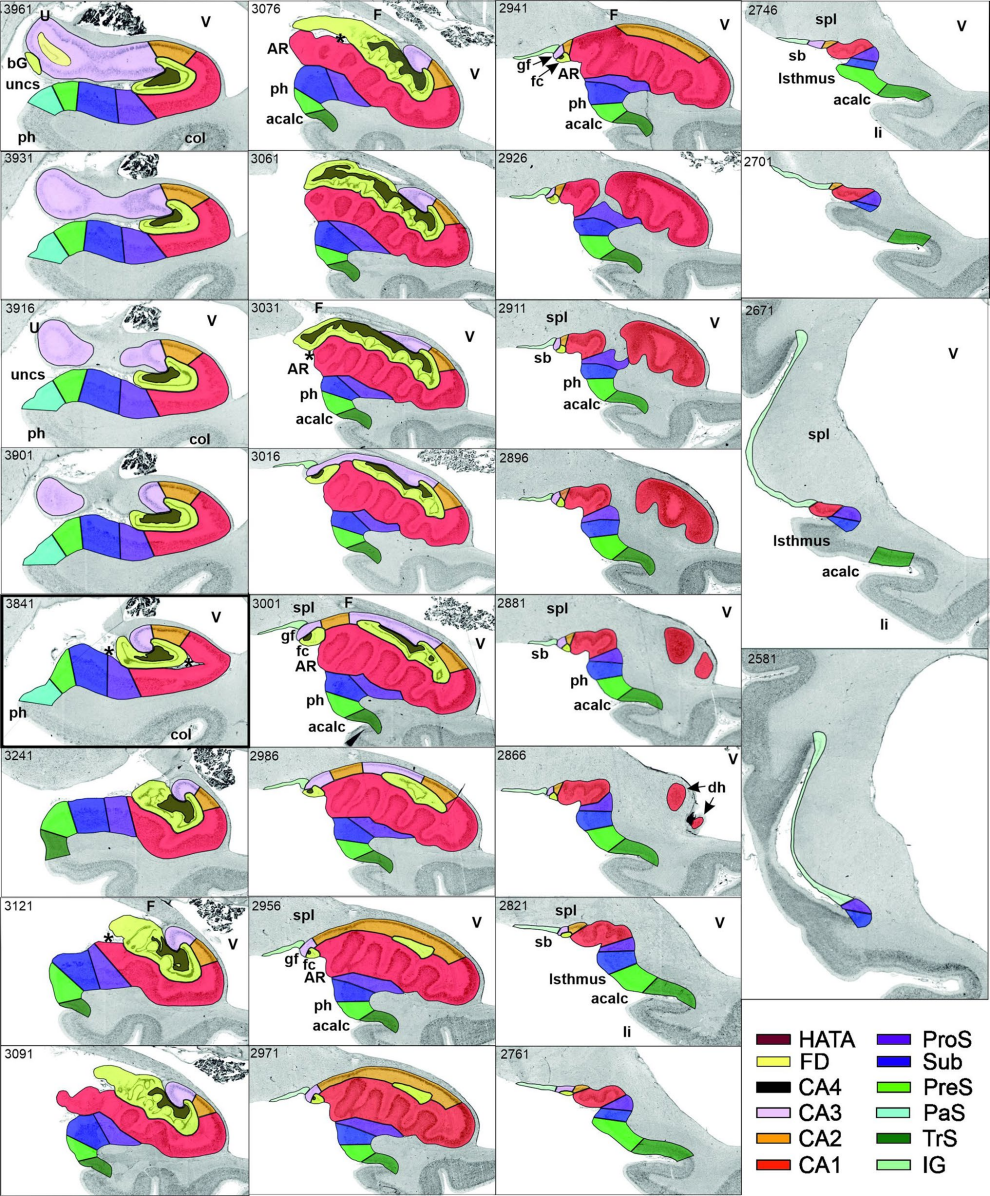
Successive coronal sections from brain B04 showing the rostro-caudal sequence of regions within the hippocampal formation and their relation to macroanatomic landmarks. The distance between sections (in µm) can be determined by calculating the differences between two section numbers (specified in the top left corner of each panel) and multiplying this result by 20 (the thickness of a single section). Sections 4561–3901 belong to the hippocampal head, the hippocampal body is represented by an exemplary section (3841; thick black frame), and sections 3241–2581 are part of the hippocampal tail, which can be further divided into initial (sections 3241–3076), middle (sections 3001–2926) and terminal (sections 2896–2581) segments, as designated by Duvernoy (1988). Note that sections 3061–3016 reveal features of both the initial and middle segments. Note also, that the subicular complex is found throughout the hippocampal head. Each cytoarchitectonic region is marked on each section by its respective color as specified at the bottom right of the figure. *acalc* anterior calcarine sulcus, *amb* ambiens gyrus, *AR* gyrus of Andreas Retzius, *bG* band of Giacomini, *CA1–4* regions 1 to 4 of the cornu Ammonis, *col* collateral sulcus, *dh* digitationes hippocampi, *F* fornix, *fc* fasciola cinerea, *FD* fascia dentata, *gf* fasciolar gyrus, *HATA* hippocampal-amygdaloid transition area, *IG* indusium griseum, *li* superior lingual gyrus, *PaS* parasubiculum, *ph* parahippocampal gyrus, *PreS* presubiculum, *ProS* prosubiculum, *sb* subsplenial gyrus, *spl* splenium, *Sub* subiculum, *TrS* transsubiculum, *U* uncal apex, *unc* uncinate gyrus, *uncs* uncinate sulcus, *V* ventricle. Asterisk indicates the position of the hippocampal fissure

Based on cyto- and receptorarchitectonic criteria, we delineated five regions within the hippocampus (FD, CA4, CA3, CA2, CA1) and four within the subicular complex (ProS, Sub, PreS and PaS; Fig. 3). In addition to the borders between these regions, in some cases we could also identify subregions with distinct receptorarchitectonic features.

**Fig. 4:**
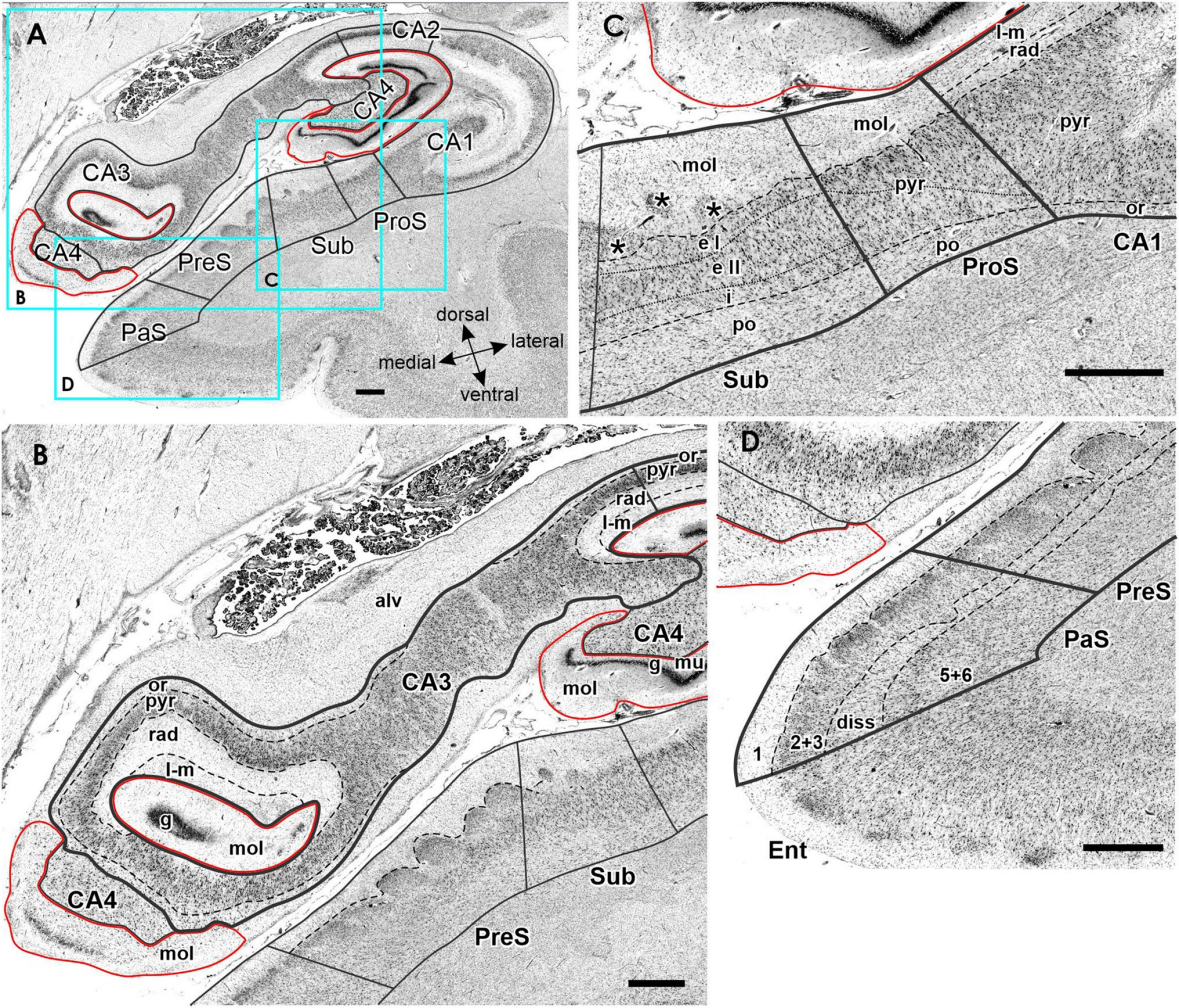
Cytoarchitecture of the hippocampal head and adjacent subicular complex. **a** Overview of an exemplary section comparable in its rostro-caudal position to section 3961 in Fig. 3. Cutouts (position highlighted by blue frames in **a** demonstrate differences in cytoarchitecture between **b** hippocampal regions FD, CA4 and CA3, **c** CA1, ProS and Sub, as well as **d** PreS and PaS. The dotted line in ProS highlights the gradual decrease in the width of the outer pyramidal sublayer (which contains pyramids typical of the CA region) and concomitant increase in the width of the inner pyramidal sublayer (which contains typical subicular pyramids). The dotted lines in Sub separate the two external sublayers (e I and e II) of the pyramidal layer from its internal (i) sublayer. Asterisks highlight the clusters of layer 2 cells from PreS which invade Sub. *1* layer 1 (molecular layer), *2* + *3* layers 2 and 3 (pyramidal layers), *5* + *6* layers 5 (parvocellular layer) and 6 (polymorph layer), *alv* alveum, *CA1–CA4* regions 1–4 of the cornu Ammonis, *diss* dissecans layer, *e I* external sublayer I of the pyramidal layer, *e II* external sublayer II of the pyramidal layer, *Ent* entorhinal cortex, *FD* fascia dentata, *g* granular layer, *i* internal sublayer of the pyramidal layer, *l-m* lacunosum-molecular layer, *mol* molecular layer, *mu* multiform layer, *or* oriens layer, *PaS* parasubiculum, *po* polymorph layer, *PreS* presubiculum, *ProS* prosubiculum, *pyr* pyramidal layer, *rad* radiatum layer. Scale bar 1 mm

**Fig. 5:**
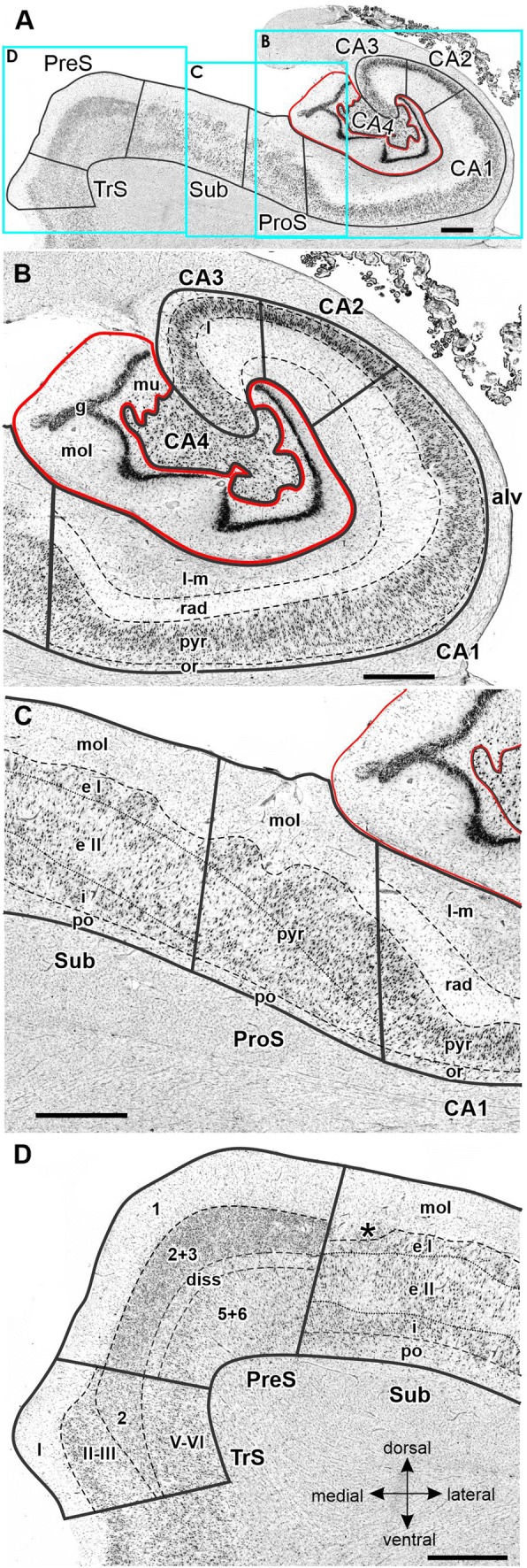
Cytoarchitecture of the hippocampal body and adjacent subicular complex. **a** Overview of an exemplary section comparable to the sectioning level *3241* in Fig. 3. Cutouts (position highlighted by blue frames in **a** demonstrate differences in cytoarchitecture between **b** hippocampal regions FD, CA4, CA3, CA2 and CA1, **d** subicular regions Sub, PreS and TrS. Asterisk highlights a cluster of layer 2 cells from PreS which invades Sub. Roman numerals indicate isocortical layers. *1* layer 1 (molecular layer), *2* + *3* layers 2 and 3 (pyramidal layers), *5* + *6* layers 5 (parvocellular layer) and 6 (polymorph layer), *alv* alveum, *CA1–CA4* regions 1–4 of the cornu Ammonis, *diss* dissecans layer, *g* granular layer, *l* lucidum layer (note, that its border with the radiatum layer has not been indicated, because it is not revealed by the silver cell body staining), *l-m* lacunosum-molecular layer, *mol* molecular layer, *mu* multiform layer, *or* oriens layer, *po* polymorph layer, *PreS* presubiculum, *ProS* prosubiculum, *pyr* pyramidal layer, *rad* radiatum layer, *Sub* subiculum, *TrS* transsubiculum. Scale bars 1 mm

**Fig. 6:**
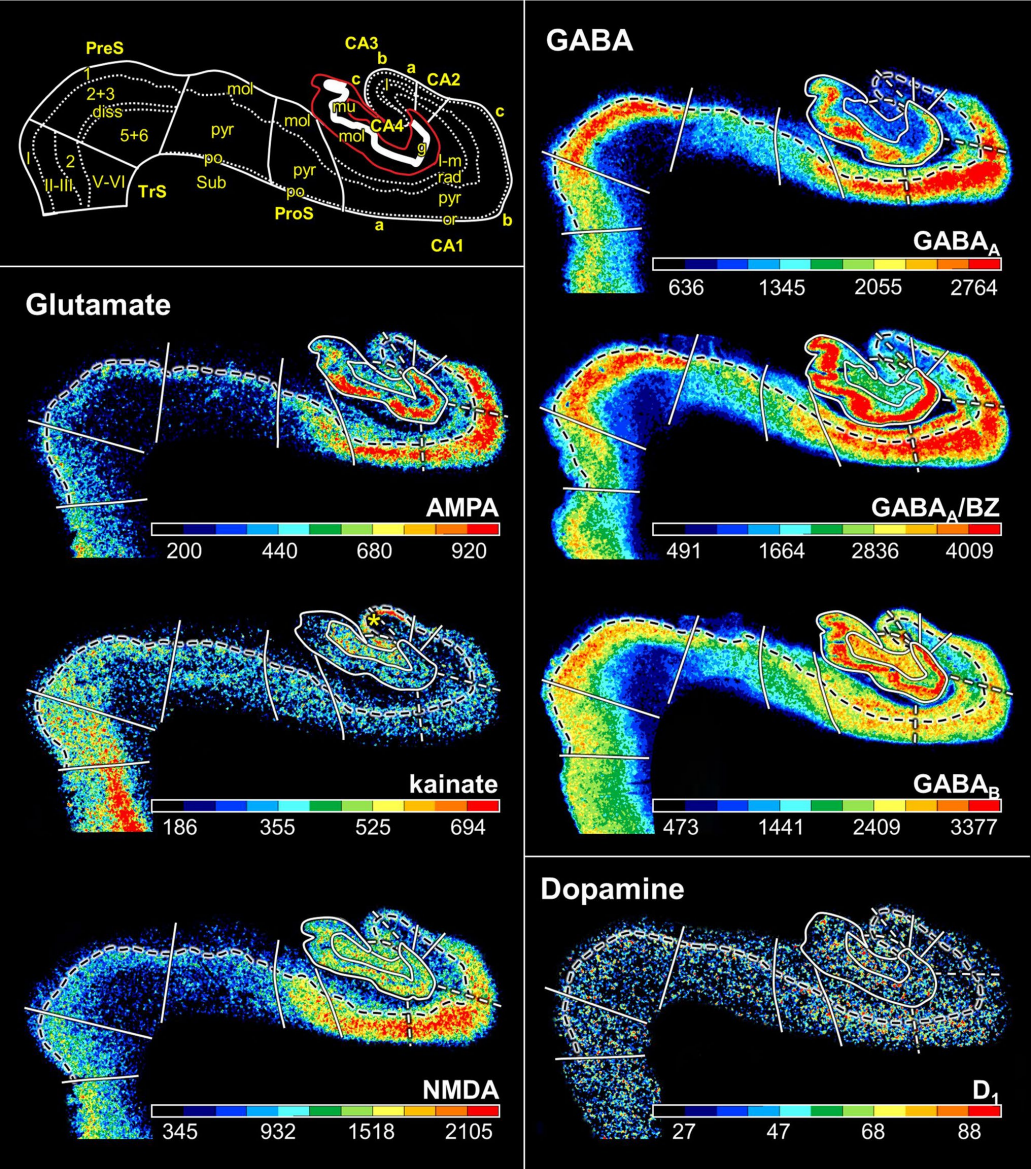

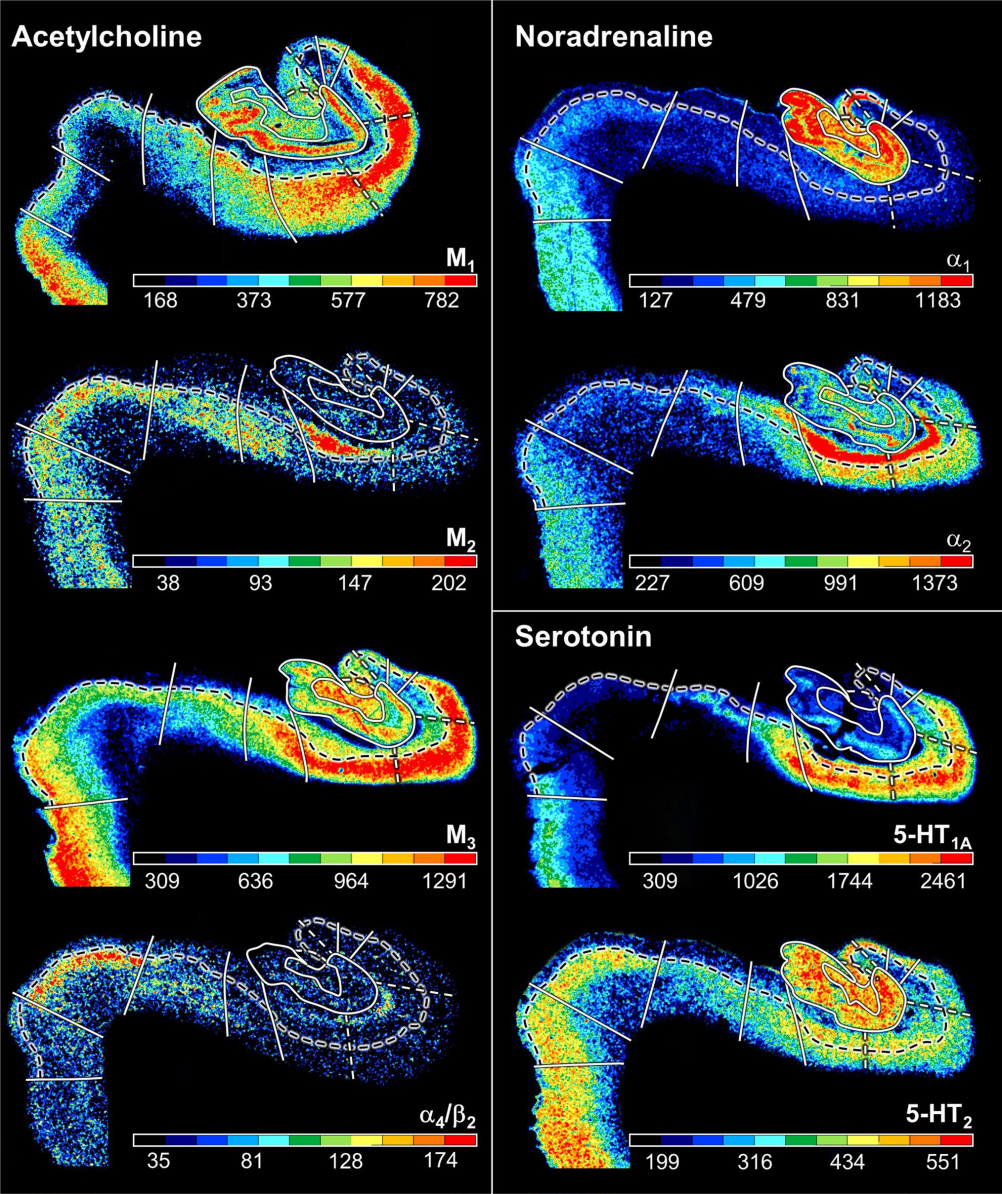
Distribution of receptors for glutamate, GABA, dopamine, acetylcholine, noradrenaline and serotonin in the hippocampal formation visualized in serial sections through the hippocampal body at a rostro-caudal level comparable to section 3241 in Fig. 3. The schematic drawing depicts cytoarchitectonic layers (dotted lines) and regions (continuous lines) as identified in an adjacent section processed for silver cell body staining. The fascia dentata is circumscribed in red. Note, that only the border between the pyramidal and its adjacent superficial layer (lucidum in CA3, radiatum in CA2 and CA1, molecular in ProS, Sub and PreS, layer I in TrS) is highlighted in the autoradiographs (dashed black lines). White dashed lines in the autoradiographs indicate the borders between the CA1a (adjacent to ProS), CA1b, and CA1c (adjacent to CA2) subdivisions of the CA1 region, as well as between the CA3a (adjacent to CA2), CA3b, and CA3c (adjacent to CA4) subdivisions of CA3. Color bars code for receptor densities in fmol/mg protein. Asterisk highlights the high density of kainate receptors in the lucidum layer of CA3. Roman numerals indicate isocortical layers. *1* layer 1 (molecular layer), *2* + *3* layers 2 and 3 (pyramidal layers), *5* + *6* layers 5 (parvocellular layer) and 6 (polymorph layer), *a, b, c* subdivisions of areas CA1 and CA3, *CA1–CA4* regions 1–4 of the cornu Ammonis, *diss* dissecans layer, *g* granular layer, *l* lucidum layer (note, that its border with the radiatum layer has not been indicated, because it is not revealed by the silver cell body staining), *l-m* lacunosum-molecular layer, *mol* molecular layer, *mu* multiform layer, *or* oriens layer, *po* polymorph layer, *PreS* presubiculum, *ProS* prosubiculum, *pyr* pyramidal layer, *rad* radiatum layer, *Sub* subiculum, *TrS* transsubiculum

**Fig. 7:**
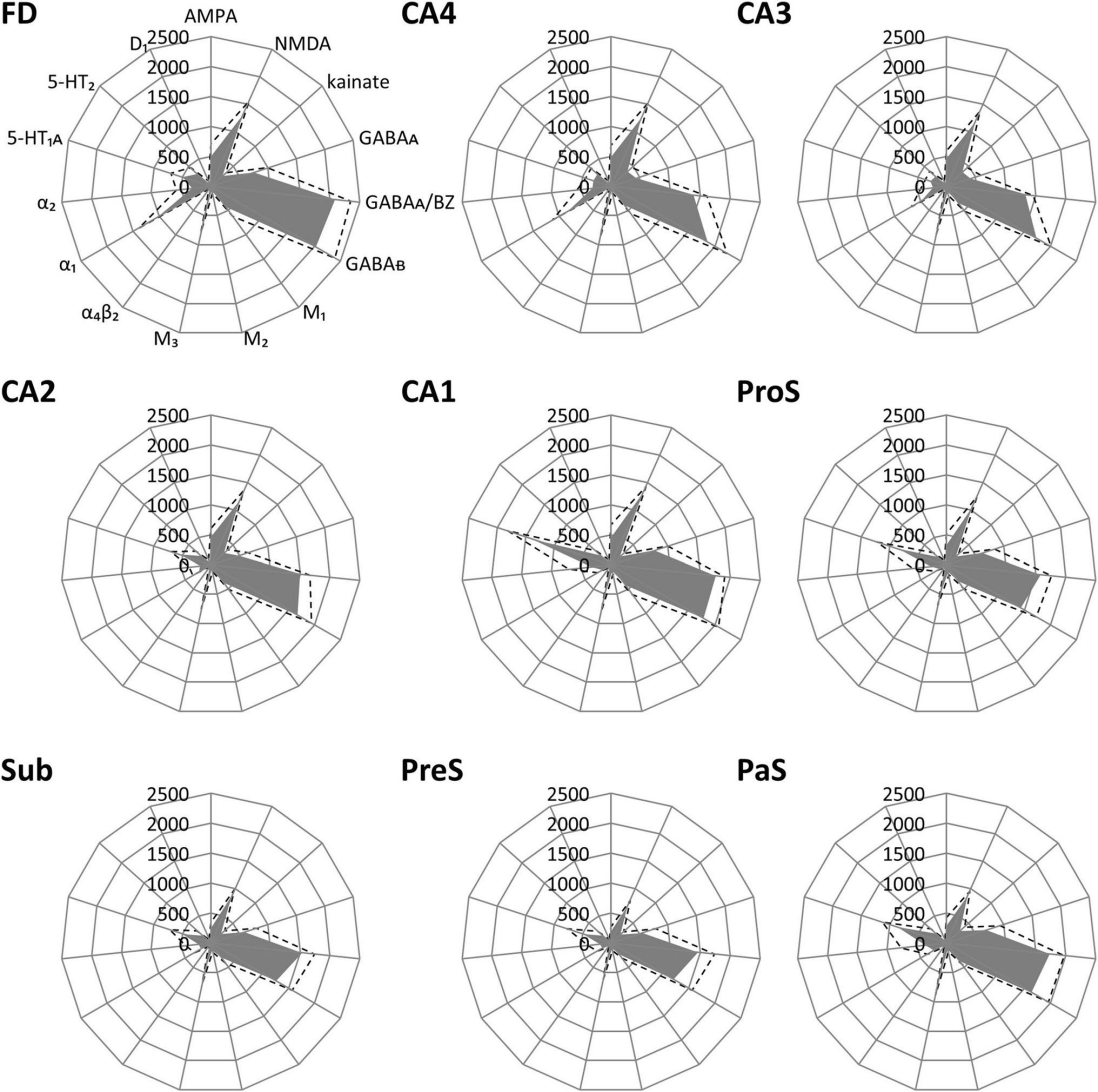
Receptor fingerprints of hippocampal regions depicting the absolute densities (in fmol/mg protein) of 15 receptors. The positions of the different receptor types are identical in all polar plots, and specified in the polar plot for the *fascia dentata* (FD). The grey area represents the mean absolute receptor densities, and dashed lines indicate the standard error of the mean. *CA1–CA4* sectors 1–4 of the *cornu Ammonis*, *FD fascia dentata, PaS* parasubiculum, *PreS* presubiculum, *ProS* prosubiculum, *Sub* subiculum

FD, located on the DG together with the CA4 region, is the most medial of the regions defined within the hippocampus proper, although CA1 is the first one to appear when moving from rostral to caudal (Supplementary Table 1; Fig. 3, sections 4486 and 4081). The smaller and less densely packed cells in the multiform layer of FD enable its separation from CA4, where neurons show a more dense packing (Figs. 4a, b, 5a, b). Furthermore, cell bodies tend to form clusters in CA4, but not in the multiform layer of the FD. The border between FD and CA4 was also clearly revealed by differences in the densities of receptors for glutamate, GABA, acetylcholine and noradrenaline (Fig. 6). The multiform layer contained lower AMPA, NMDA, kainate, GABA_B_, M_1_ and M_3_ receptor densities and GABA_A_ associated benzodiazepine (GABA_A_/BZ) binding site concentrations than did the CA4 region. Conversely, α_1_ receptor densities were higher in the multiform layer than in CA4. Furthermore, AMPA, GABA_A_, M_1_, M_3_ and α_2_ receptors are heterogeneously distributed throughout the molecular layer of the FD, with higher densities in its outer than inner portions (Fig. 6). These differences in receptor distribution patterns between FD and CA4 are also reflected in differently shaped and sized receptor fingerprints, with that of CA4 being smaller (Fig. 7).

CA4, one of the smallest regions of the hippocampal formation (Table 3), is flanked by FD and CA3 (Fig. 3). The cytoarchitectonic border between CA3 and CA4 is characterized by the presence of the oriens, pyramidal, lucidum, and radiatum-lacunosum-molecular layers in CA3, but only of the pyramidal layer in CA4 (Figs. 4b, 5b). This delineation is further supported by differences in the densities of NMDA, M_1_ and α_2_ receptors, which were considerably lower in the pyramidal layer of CA3 than in CA4 (Fig. 5). The receptor fingerprint of CA3 is smaller than that of CA4, although they are both quite similar in shape (Fig. 7).

**Table 3:** Volume measurements in the hippocampal formation

	Total	Left	Right	Male	Female
FD	806 ± 145	800 ± 153	811 ± 145	760 ± 184	851 ± 77
CA4	137 ± 30	136 ± 31	138 ± 30	131 ± 37	142 ± 22
CA3	248 ± 38	247 ± 41	249 ± 37	225 ± 28	271 ± 34
CA2	183 ± 29	180 ± 33	187 ± 25	167 ± 23	200 ± 24
CA1	1437 ± 239	1419 ± 228	1456 ± 261	1311 ± 252	1563 ± 148
ProS	377 ± 61	380 ± 65	373 ± 60	321 ± 22	432 ± 25
Sub	531 ± 106	529 ± 113	533 ± 104	475 ± 121	587 ± 45
PreS	345 ± 71	337 ± 70	353 ± 75	314 ± 66	376 ± 64
PaS	123 ± 39	126 ± 41	119 ± 39	94 ± 11	151 ± 36
TrS	115 ± 29	112 ± 24	118 ± 35	120 ± 22	110 ± 36

For each region, its mean volume (± s.d.) on the right and left hemisphere, and in male and female brains, as well as its mean total volume are given in mm^3^. Volumes are given after individual correction for shrinkage during histological processing.

**Fig. 8:**
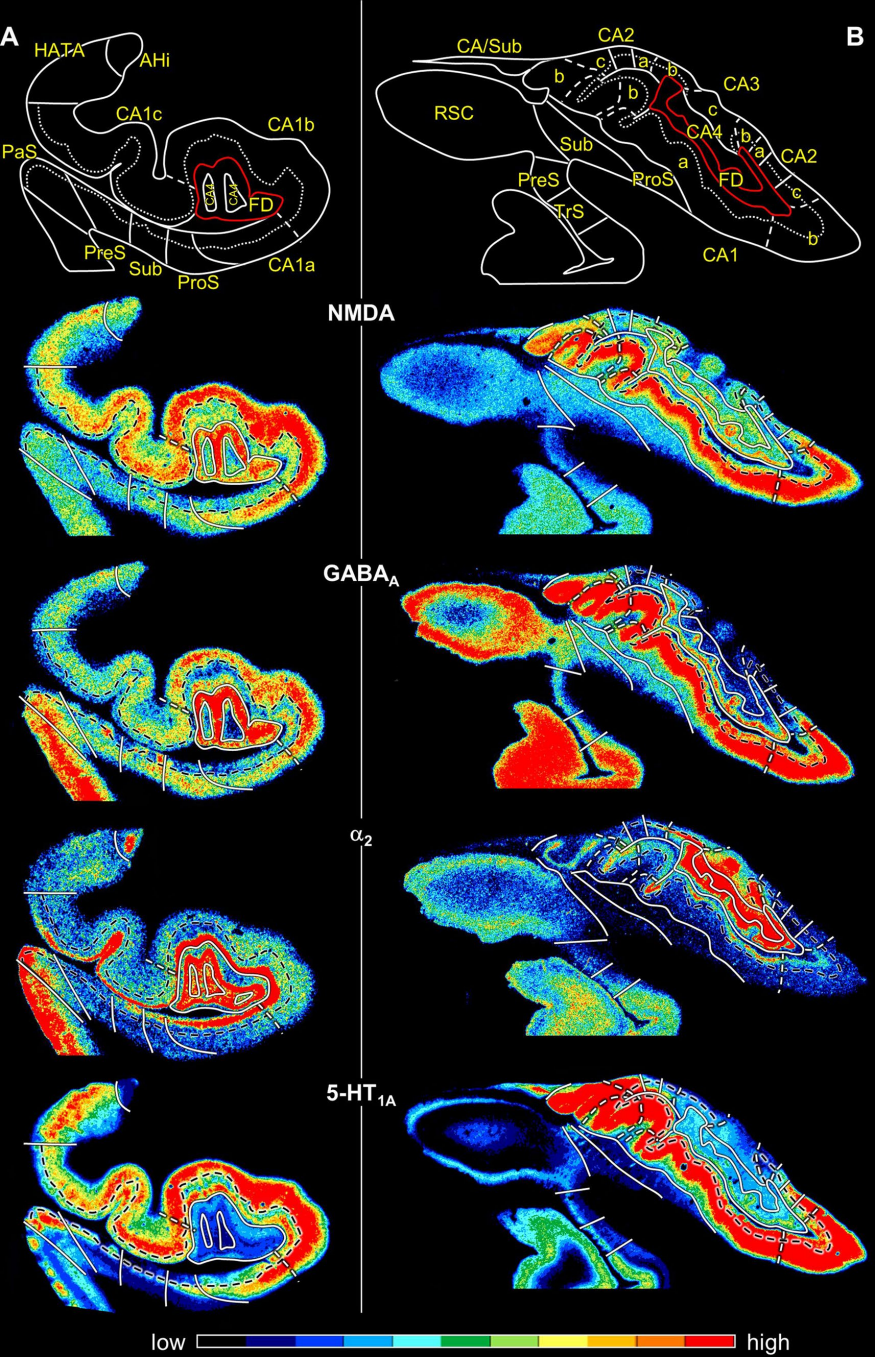
Exemplary autoradiographs depicting the distribution of the glutamate NMDA, GABA_A_, noradrenaline α_2_ and serotonin 5-HT_1A_ receptors at two different rostro-caudal levels of thee hippocampal formation. **a** Sections through the hippocampal head at a rostro-caudal level comparable to section 4261 in Fig. 3. **b** Sections through the hippocampal tail at a level roughly comparable to section 3016 in Fig. 3. Roman numerals indicate isocortical layers. *1* layer 1 (molecular layer), *2* + *3* layers 2 and 3 (pyramidal layers), *5* + *6* layers 5 (parvocellular layer) and 6 (polymorph layer), *a, b, c* subdivisions of areas CA1 and CA3, *AHi* amygdalohippocampal transition area, *CA1–CA4* regions 1 to 4 of the cornu Ammonis, *CA/Sub* rudimental hippocampus and subiculum (caudalmost portion of the subsplenial indusium griseum), *diss* dissecans layer, *g* granular layer, *l* lucidum layer (note, that its border with the radiatum layer has not been indicated, because it is not revealed by the silver cell body staining), *l-m* lacunosum-molecular layer, *mol* molecular layer, *mu* multiform layer, *or* oriens layer, *po* polymorph layer, *PreS* presubiculum, *ProS* prosubiculum, *pyr* pyramidal layer, *rad* radiatum layer, *RSC* retrosplenial cortex, *Sub* subiculum, *TrS* transsubiculum

CA3 is the only region in which the lucidum layer, as clearly revealed by high kainate and α_1_ receptor densities (asterisk in Fig. 6), is present. The border between CA3 and CA2 could also be defined cytoarchitectonically by differences in the arrangement of cell bodies within the pyramidal layer: they are homogeneously distributed in CA3, but form a superficial, more densely packed sublayer, and a deep, less densely packed sublayer in CA2 (Fig. 5b). The border between CA3 and CA2 is also revealed by differences in the densities of GABA_A_, GABA_B_, M_1_, M_3_ and 5-HT_1A_ receptors, as well as of GABA_A_/BZ binding sites, which were lower in CA3 than in CA2. These differences are particularly obvious in the pyramidal layer, but are also reflected in receptor fingerprints. The CA3 fingerprint has a more pronounced difference in the densities of GABA_A_/BZ binding sites and GABA_B_ receptors than that of CA2, which presents a clear peak at the level of the 5-HT_1A_ receptors (Fig. 7). The opposite holds true for 5-HT_2_ receptor densities (Fig. 6). Interestingly, receptor distribution patterns reveal the existence of three subdivisions within CA3: CA3a (adjacent to CA2), CA3b and CA3c (adjacent to CA4). Densities of NMDA, AMPA, GABA_B_, M_1_, M_3_, and α_2_ receptors as well as of GABA_A_/BZ binding sites are lowest in the radiatum and lacunosum-molecular layers of CA3a and highest in those of CA3c (Figs. 6, 8).

CA2, also a relatively small region (Table 3), is located between the CA1 and CA3 regions (Fig. 3). The border between CA2 and CA1 was characterized by a narrower and more densely packed pyramidal layer in CA2 than in CA1 (Fig. 5). This finding matches differences in the densities of AMPA, NMDA, GABA_A_, M_1_, M_3_, α_2_, and 5-HT_1A_ receptors as well as of GABA_A_/BZ binding sites, which were higher in CA1 than in CA2 (Fig. 6). The differences were particularly striking in the pyramidal layer, though they were also obvious in the radiatum and lacunosum-molecular layers, and in the receptor fingerprints depicting the densities of each of the 15 receptors averaged over all layers of CA1 and CA2 (Fig. 7).

CA1 is the largest region in the hippocampal formation (Table 3), and the first part of the cornu Ammonis to appear when moving from rostral to caudal (Supplementary Table 1; Fig. 3 section 4486). It is bordered medially by the subicular complex (i.e., by its ProS region), and dorso-laterally by CA2. The most caudal portion of CA1 is located on the Retzius gyrus, beneath the splenium of the corpus callosum. Cytoarchitectonically, the border between CA1 and ProS is characterized by the abrupt disappearance of the radiatum layer and the gradual appearance of large subicular-like pyramids in the deeper portion of the pyramidal layer of ProS, as well as of a rudimentary polymorph layer directly abutting the white matter (Figs. 4c, 5c). The border between CA1 and ProS was also highlighted by conspicuous changes in the densities of many receptors (Fig. 6). The pyramidal layer of ProS, even in its superficial portion, which contains CA-like pyramids, contained lower AMPA, NMDA, GABA_A_, M_2_, α_2_, and 5-HT_1A_ receptor densities as well as GABA_A_/BZ binding sites, than that of CA1. Additionally, the molecular layer of CA1 contained higher nicotinic α_4_/α_2_ receptor densities than that of ProS. The most conspicuous differences between the fingerprints of CA1 and ProS are the pronounced 5-HT_1A_ receptor peak in the former region, and the reversal of the balance of GABA_A_/BZ binding site and GABA_B_ receptors in ProS (Fig. 7). Indeed, whereas in CA1–CA4 the densities of GABA_B_ receptors were higher than those of GABA_A_/BZ binding sites, the opposite holds true for regions of the subicular complex.

Changes in receptor distribution patterns also highlighted the existence of three subdivisions within CA1, which were particularly obvious in the hippocampal body (Fig. 6), though not restricted to this rostro-caudal segment (Fig. 8): CA1a (bordering ProS), CA1b, and CA1c (adjacent to CA2). Interestingly, these differences were pronounced in the radiatum and lacunosum-moleculare layers, but somewhat gradual in the pyramidal layer, and were not visible in sections processed for the visualization of cell bodies. CA1a and CA1b could be distinguished by the higher M_2_ and 5-HT_1A_ densities in the radiatum layer, lower α_4_/α_2_ densities in the lacunosum-molecular layer, and lower NMDA and GAABA_A_ densities in the pyramidal layer of CA1a than in the corresponding layers of CA1b. CA1b and CA1c differed in their NMDA, GABA_A_, α_2_ and 5-HT_1A_ receptor densities, which were higher in the pyramidal layer of the former subregion. Additionally, the radiatum layer of CA1b contained higher kainate, NMDA, GABA_A_ GABA_B_, M_3_, α_1_, α_2_, 5-HT_1A_ and 5-HT_2_ receptor densities, as well as GABA_A_/BZ binding site densities than that of CA1c.

The most lateral portion of the subicular complex, i.e., area ProS (Supplementary Table 2), is characterized by the gradual disappearance of the pyramidal cells typical of the CA region, which in ProS form a superficial cell dense sublayer adjacent to the molecular layer, accompanied by the gradual appearance of a deeper cell layer containing large subicular-like pyramids (Figs. 4c, 5c). The border between ProS and Sub was cytoarchitectonically characterized by the existence of a typical polymorph layer and differentiation of the pyramidal layer in the latter region (Figs. 4c, 5c): an external pyramidal sublayer, where pyramids tend to form clusters, is followed by an internal pyramidal sublayer, where they present a radial arrangement and by a cell sparse internal sublayer populated by polymorph neurons (sublayer i). Furthermore, the molecular layer of Sub presented isolated clusters of presubicular layer 2 cells, which could be identified, because they contained smaller and more densely packed pyramids than Sub clusters. At the receptor level, differences between ProS and Sub were mostly restricted to external sublayer of the pyramidal layer, where densities of AMPA, NMDA, GABA_B_, 5-HT_1A_, and 5-HT_2_ receptors were higher in ProS than in Sub (Fig. 6). Additionally, NMDA, GABA_A_, GABA_B_, and α_2_ receptor densities as well as GABA_A_/BZ binding site concentrations were higher in the molecular layer of ProS than in that of Sub. Finally, nicotinic α_4_/β_2_ receptor densities were consistently higher in Sub than in ProS. These differences were also reflected at the mean regional level, where Sub presented a smaller fingerprint than ProS (Fig. 7).

Sub, the largest of the regions defined within the subicular complex (Table 3), and the first of the regions within the hippocampal formation to appear when moving from rostral to caudal (Fig. 3; Supplementary Tables 1 and 2), is followed medially by PreS. The border between these two regions is clearly visible due to the appearance in PreS of the dissecans layer, a cell sparse layer separating the external (layers 1–3) and internal layers (layers 5 and 6; Figs. 4, 5). The external layers of PreS contain higher kainate, GABA_A_, GABA_B_, α_4_/β_2_, α_1_, 5-HT_1A_ and 5-HT_2_ receptor densities as well as GABA_A_/BZ binding site concentrations than do the superficial layers of Sub (Fig. 6). Additionally, the internal layers of PrS contained lower kainate, NMDA, GABA_A_, GABA_B_, M_1_, M_3_, α_4_/β_2_, α_1_ and 5-HT_2_ receptor densities as well as GABA_A_/BZ binding site concentrations than did the deeper layers of Sub.

The cytoarchitectonic border between PreS and PaS was characterized by an increase in the size and packing density of neurons in the external layers of PaS (Fig. 4d). These changes are paralleled by higher kainate, GABA_A_, and M_3_ receptor densities as well as GABA_A_/BZ binding site concentrations, but lower M_2_ receptor densities in PaS than in PreS (Fig. 8), and by a smaller receptor fingerprint in PresS than in PaS (Fig. 7). PaS is the most external of the regions defined within the hippocampal formation, and borders the entorhinal cortex (Fig. 3; Supplementary Table 2). Although PaS was always found in the hippocampal head, it only extended into the caudal portion of the hippocampal tail in two of the examined brains (cases #7 and #13; Fig. 11). In the remaining eight brains PaS encroached on the hippocampal body to different extents, but did not reach its tail.

PaS was replaced caudally by the transsubiculum (TrS; Fig. 3), a periallocortical area located between PreS and area BA35, which displays cytoarchitectonic layers typical of both these areas. Thus, TrS has a conspicuous parvocellular layer 2, though slightly less cell dense than that of PreS. Furthermore, the gradual decrease in width of layer 2 of TrS was concomitant with the appearance of a more superficially located sublayer containing small pyramids resembling those present in isocortical layers II and III. The dissecans layer, a hallmark of PreS, is no longer visible in TrS, and the subicular-like pyramids present in layers 5 and 6 of PreS are replaced by cell bodies of different shapes. Thus, the deepest layer of TrS resembles an isocortical multiform layer VI (Fig. 5d). The superficial layers of TrS contain higher AMPA, kainate, M_1_, M_3_, α_1_, α_2_ and 5-HT_1A_ receptor densities but lower GABAergic, M_2_ and α_4_/β_2_ concentrations than do those of PreS (Fig. 6).

This multimodal analysis resulted in the definition of nine cyto- and receptorarchitectonically distinct regions (i.e., FD, CA4, CA3, CA2, CA1, ProS, Sub, PreS, and PaS) for which probabilistic maps were computed. Additionally, we could identify three subdivisions within both CA1 and CA3 (namely CA1a, CA1b, CA1c, CA3a, CA3b and CA3c) based solely on differences in receptor densities.

### Volumetric analysis

Hippocampal regions varied considerably in their relative sizes (Table 3). CA1 was the largest of the examined regions, with volumes ranging from 1196 mm^3^ in the left hemisphere of case #5 to 2178 mm^3^ in the right hemisphere of case #1. Conversely, TrS was the smallest region, and volumes ranged from 63 mm^3^ in the right hemisphere of case #13 to 181 mm^3^ in the right hemisphere of case #3.

There were no significant interhemispheric or gender differences in the sizes of the examined hippocampal regions, nor did the interaction between hemisphere and region reach the level of significance.

### Probabilistic maps and maximum probability maps

**Fig. 9:**
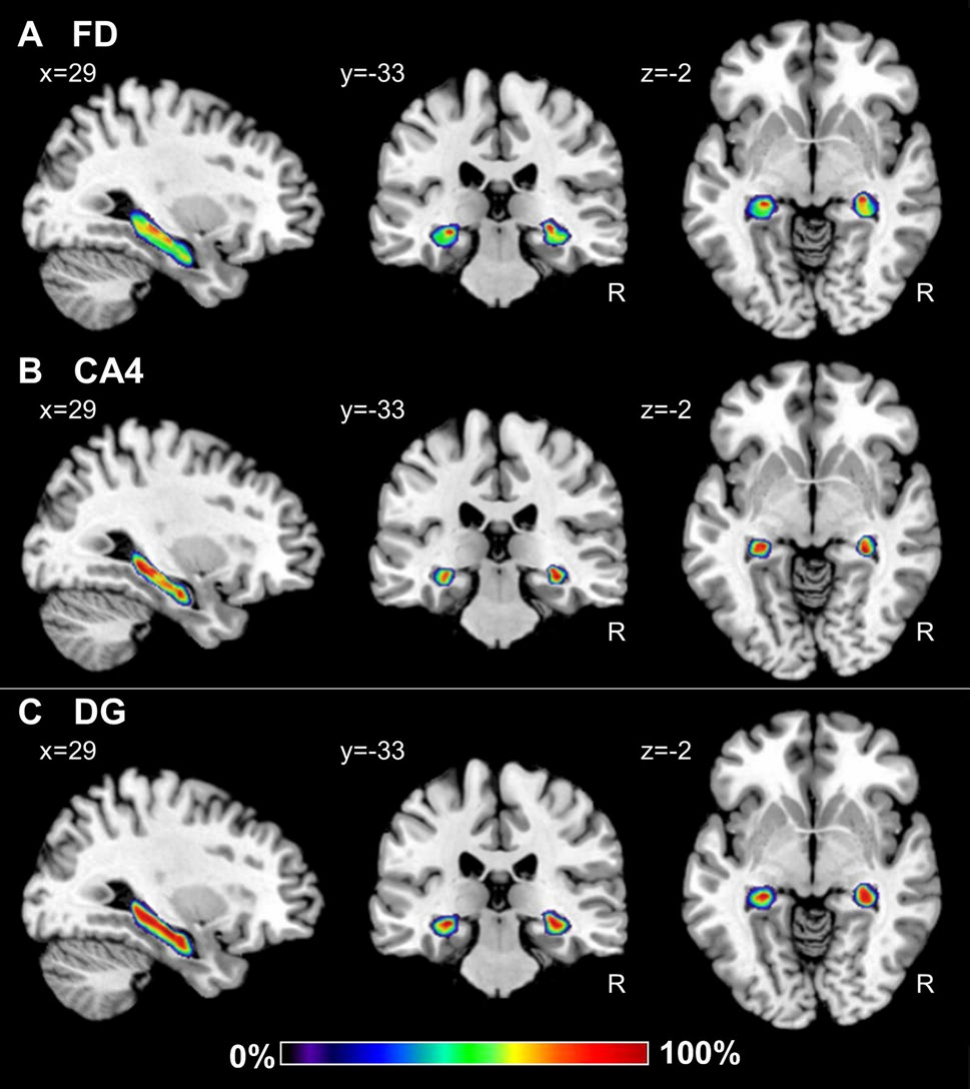
Continuous probabilistic maps of the cytoarchitectonically identifiable *fascia dentata* (FD, **a**) and CA4 region (**b**), as well as of the macroscopically identifiable dentate gyrus (DG, **c**) hippocampal and subicular regions overlayed on sagittal, coronal, and horizontal sections of the MNI single subject template (Evans et al. 2012). Stereotaxic coordinates are given in anatomical MNI space (Amunts et al. 2005). Note, that the novel workflows used for the computation of probabilistic maps result in a better reconstruction of the DG than in the previously published version of this map (compare **c** with figure 3 of Amunts et al. 2005). Color bar reflects probability of a region in a particular voxel. *R* right hemisphere

**Fig. 10:**
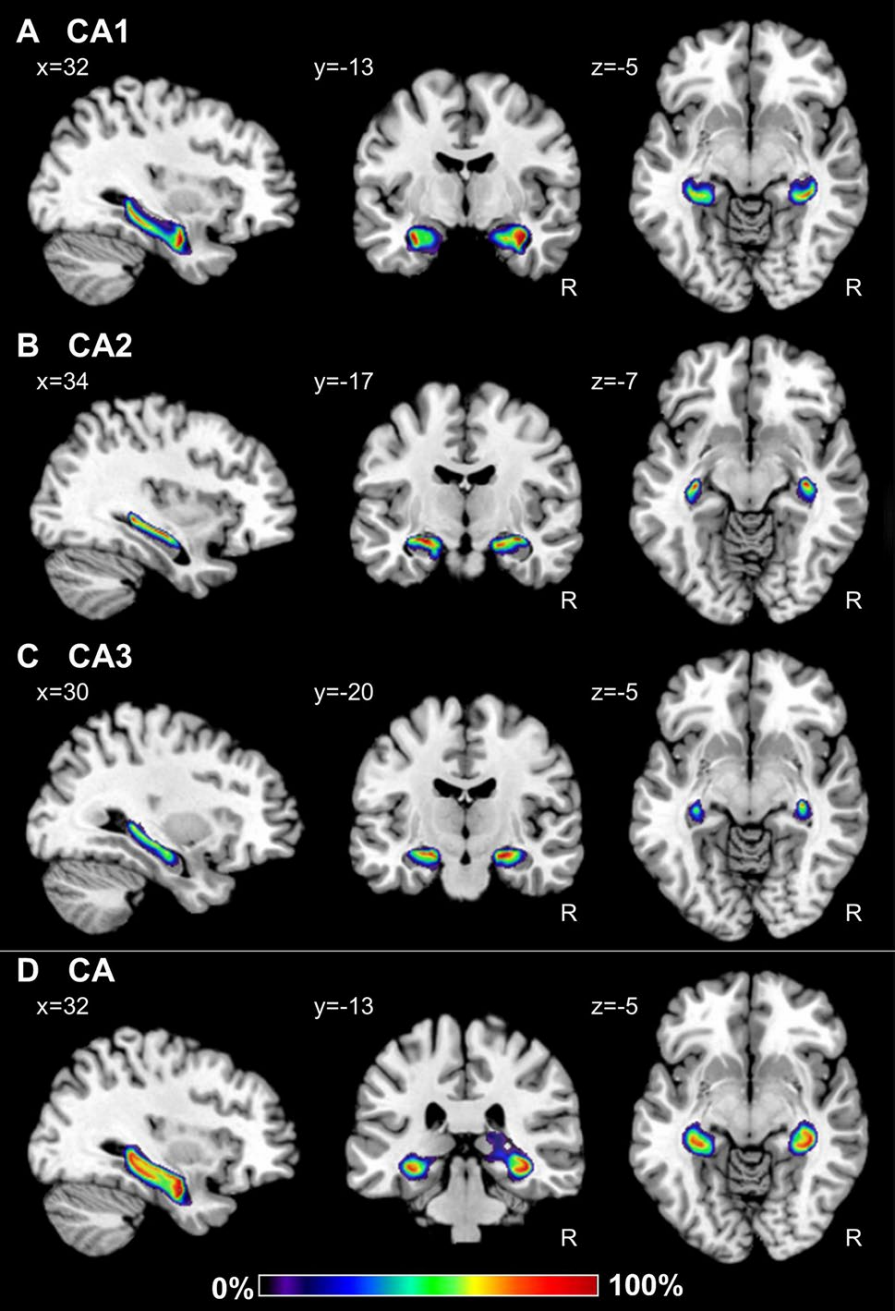
Continuous probabilistic maps of the CA1 (**a**), CA2 (**b**) and CA3 (**c**) regions, as well as of the combination of these three CA sectors (**d**) overlayed on sagittal, coronal, and horizontal sections of the MNI single subject template (Evans et al. 2012). Stereotaxic coordinates are given in anatomical MNI space (Amunts et al. 2005). Note, that the novel workflows used for the computation of probabilistic maps result in a better reconstruction of CA than in the previously published version of this map (compare **d** with figure 3 of Amunts et al. 2005). Color bar reflects probability of a region in a particular voxel. *R* right hemisphere

**Fig. 11:**
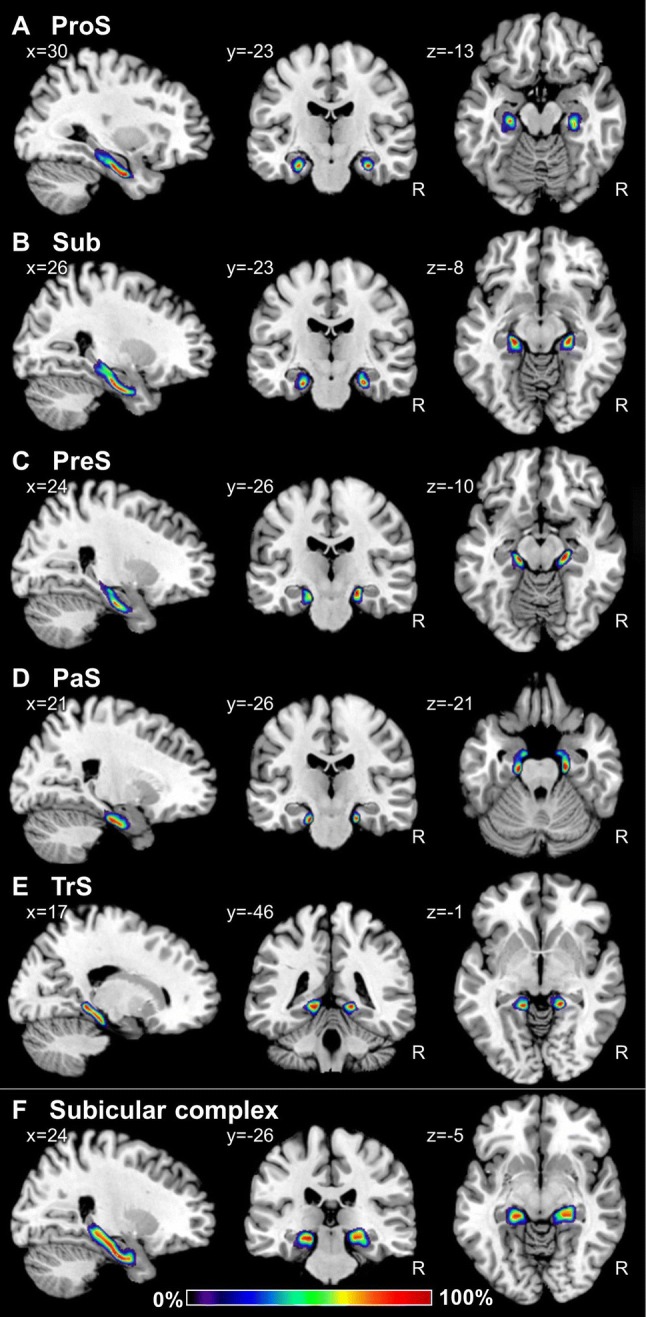
Continuous probabilistic maps of the Prosubiculum (ProS, **a**), subiculum (Sub, **b**), presubiculum (PreS, **c**), parasubiculum (PaS, **d**), and transsubiculum (TrS, **e**) regions, as well as of the combination of these cytoarchitectonic entities into the subicular complex (**f**) overlayed on sagittal, coronal, and horizontal sections of the MNI single subject template (Evans et al. 2012). Stereotaxic coordinates are given in anatomical MNI space (Amunts et al. 2005). Note, that the novel workflows used for the computation of probabilistic maps result in a better reconstruction of the subicular complex than in the previously published version of this map (compare **f** with figure 3 of Amunts et al. 2005). Color bar reflects probability of a region in a particular voxel. *R* right hemisphere

**Fig. 12:**
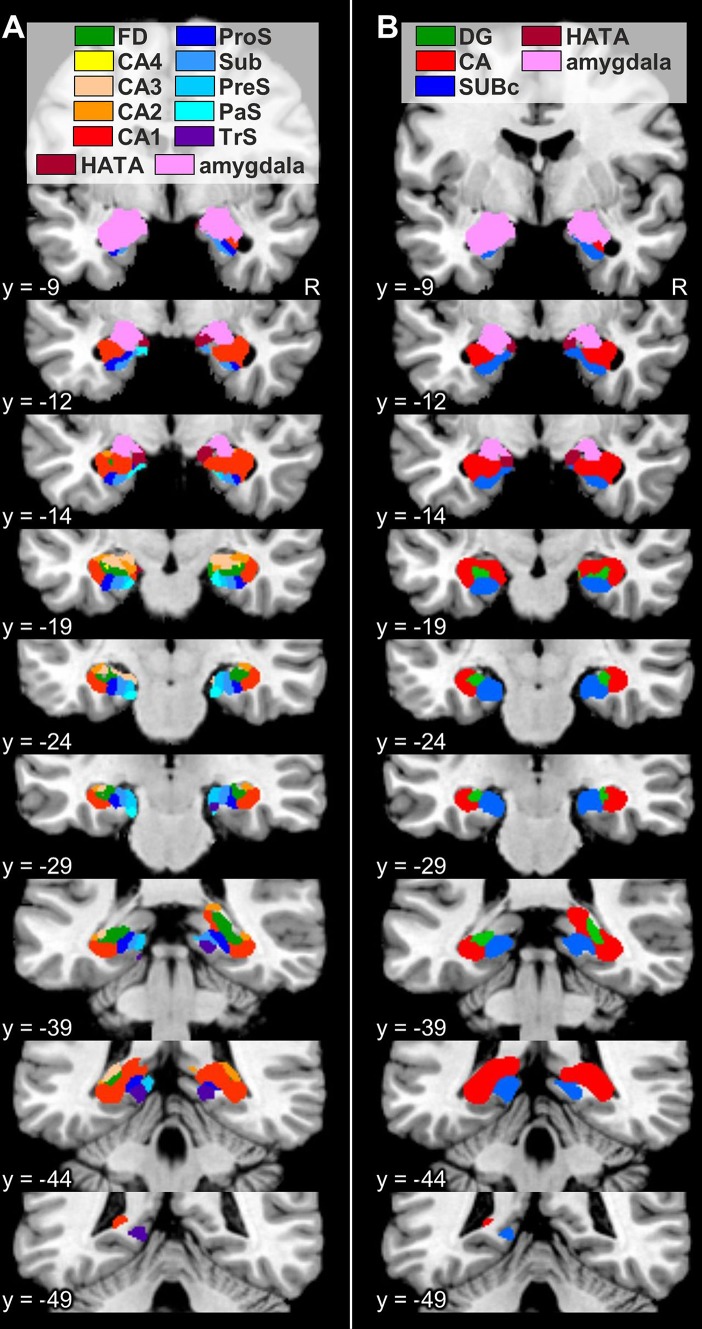
Maximum probability maps of the hippocampal formation and adjacent cortices overlayed onto coronal sections through the single subject template of the MNI space (Evans et al. 2012). Stereotaxic coordinates are given in anatomical MNI space (Amunts et al. 2005). **a** Maps of the regions of the hippocampal formation identified in the present study, as well as of the amygdala and HATA as defined in Amunts et al. (2005). **b** Maps of the regions of the hippocampal formation as defined in Amunts et al. (2005). *CA* cornu Ammonis (encompasses its regions 1–3), *CA1–4* regions 1–4 of the cornu Ammonis, *DG* dentate gyrus, *FD* fascia dentata, *HATA* hippocampal-amygdaloid transition area, *PaS* parasubiculum, *PreS* presubiculum, *ProS* prosubiculum, *R* right hemisphere, *Sub* subiculum, *SUBc* subicular complex, *TrS* transsubiculum

**Fig. 13:**
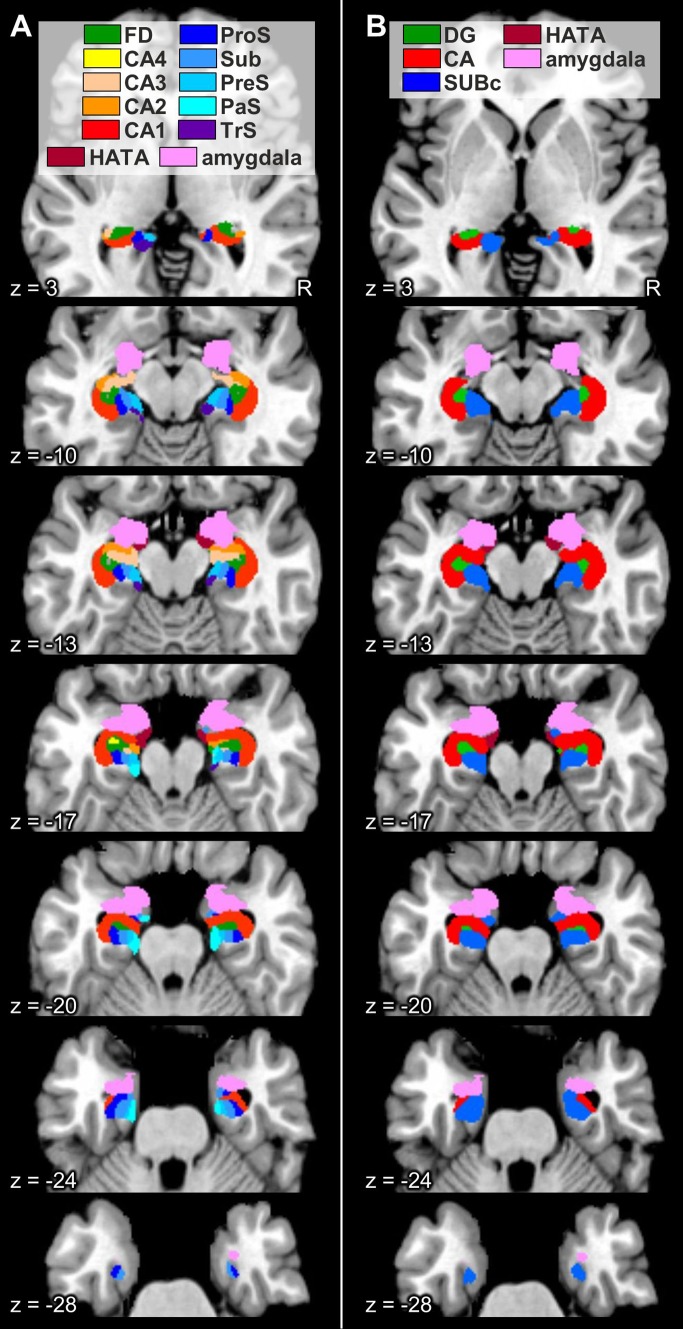
Maximum probability maps of the hippocampal formation and adjacent cortices overlayed onto horizontal sections through the single subject template of the MNI space (Evans et al. 2012). Stereotaxic coordinates are given in anatomical MNI space (Amunts et al. 2005). **a** Maps of the regions of the hippocampal formation identified in the present study, as well as of the amygdala and HATA as defined in Amunts et al. (2005). **b** Maps of the regions of the hippocampal formation as defined in Amunts et al. (2005). *CA* cornu Ammonis (encompasses its regions 1–3), *CA1–4* regions 1–4 of the cornu Ammonis, *DG* dentate gyrus, *FD* fascia dentata, *HATA* hippocampal-amygdaloid transition area, *PaS* parasubiculum, *PreS* presubiculum, *ProS* prosubiculum, *R* right hemisphere, *Sub* subiculum, *SUBc* subicular complex, *TrS* transsubiculum

**Fig. 14:**
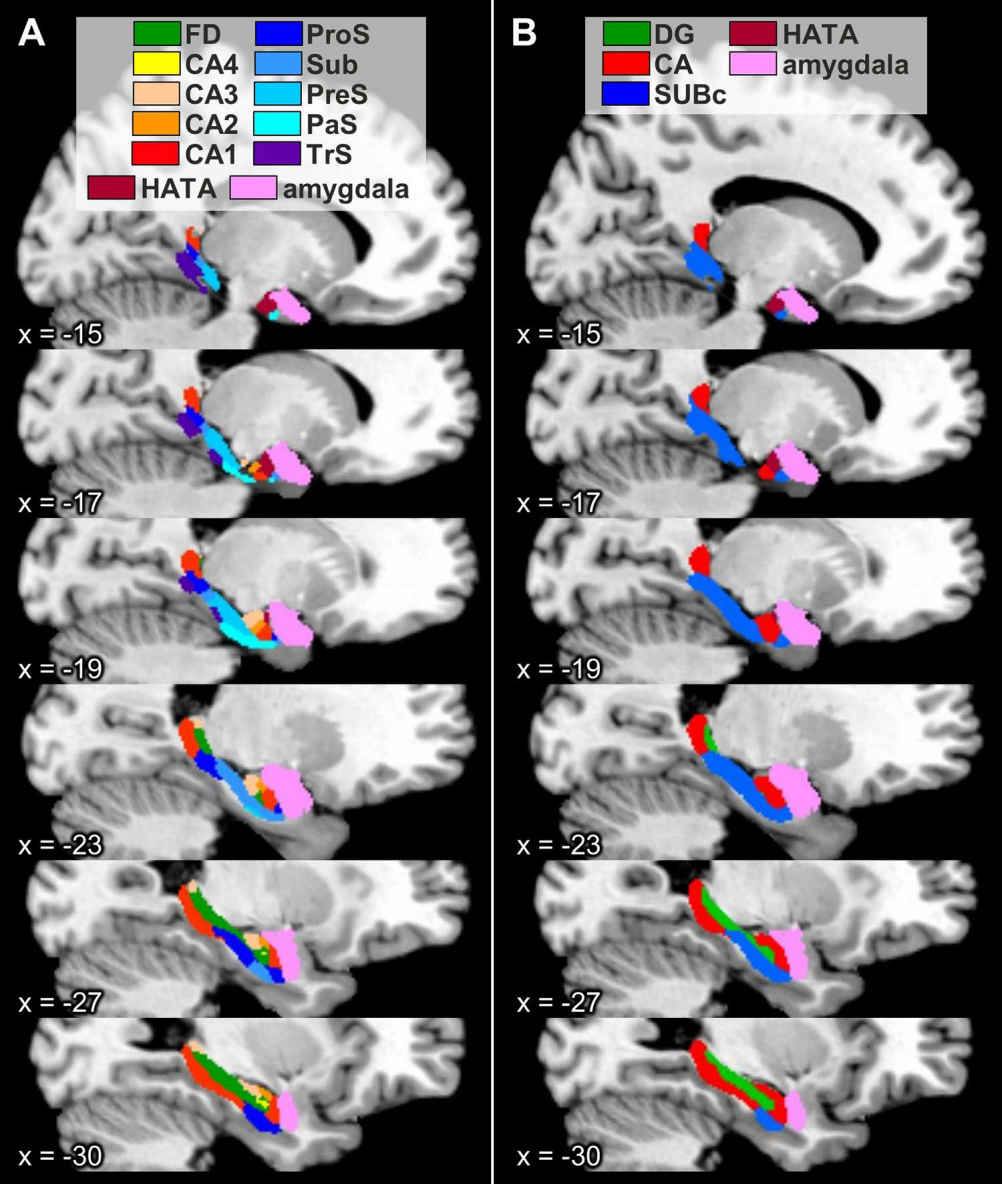
Maximum probability maps of the hippocampal formation and adjacent cortices overlayed onto sagittal sections through the single subject template of the MNI space (Evans et al. 2012). Stereotaxic coordinates are given in anatomical MNI space (Amunts et al. 2005). **a** Maps of the regions of the hippocampal formation identified in the present study, as well as of the amygdala and HATA as defined in Amunts et al. (2005). **b** Maps of the regions of the hippocampal formation as defined in Amunts et al. (2005). *CA* cornu Ammonis (encompasses its regions 1–3), *CA1–4* regions 1–4 of the cornu Ammonis, *DG* dentate gyrus, *FD* fascia dentata, *HATA* hippocampal-amygdaloid transition area, *PaS* parasubiculum, *PreS* presubiculum, *ProS* prosubiculum, *Sub* subiculum, *SUBc* subicular complex, *TrS* transsubiculum

Probability maps revealed a lower degree of topographical variability for CA regions than for the FD or those located within the subicular complex (Figs. 9, 10, 11). Thus, highest probabilities, i.e., where a particular region was present in eight or more of ten brains, were observed in a larger amount of voxels in CA, in particular in the CA1 and CA3 regions. Within the subicular complex, the greatest degree of variability was observed for PaS, whereas the largest overlap was obtained for the Sub and TrS regions. Since this degree of intersubject variability resulted in an overlap of the probability maps, we computed maximum probability maps for each region (Figs. 12, 13, 14). These maps represent a contiguous, non-overlapping parcellation of the hippocampal complex, and although they bear a close conceptual resemblance to classical brain maps, they are not a mere schematic visualization of “typical” hippocampal regions, but reflect the most likely region based on our sample of ten brains and represented in each voxel of the reference space, and provide an adequate representation of the stereotaxic location of each region in each individual brain.

## Discussion

We here applied a multimodal mapping approach to identify cyto- and receptorarchitectonically distinct regions within the human hippocampal formation and subsequently applied novel workflows for the computation of probabilistic maps resulting in a revised and more detailed version of our previous map (Amunts et al. 2005). Ten regions were evaluated: FD, CA4, CA3, CA2 and CA1 within the hippocampus proper, ProS, Sub, PreS, PaS and TrS within the subicular complex. The cytoarchitectonic probabilistic maps quantify intersubject variability in the size and extent of cyto- and receptorarchitectonically distinct regions composing the hippocampal formation and neighbouring cortex, and constitute the basis for future architectonically informed analyses of neuroimaging studies.

Variations in the densities of some receptor types enabled identification of subdivisions within the CA1 and CA3 regions (i.e., CA1a, CA1b, CA1c, CA3a, CA3b and CA3c). These subdivisions were not considered when computing probabilistic maps, because they cannot (or not reliably so) be identified in cell body stained sections (Lorente de Nó 1934). In addition, these subdivisions are very small, and cannot be presented in a meaningful way in standard reference space, which is limited to 1-mm spatial resolution. The present receptorarchitectonic analysis demonstrated, however, that these subdivisions are functionally different on a molecular level.

Multimodal mapping has proven to be a powerful tool in brain research, since differences in receptor densities not only confirm the borders of cytoarchitectonically defined areas, but also reveal subdivisions not visible in tissue processed for the visualization of cell bodies (e.g., Geyer et al. 1996, Palomero-Gallagher et al. 2008). Differences in cyto- and receptorarchitecture, with their subsequent differential responses to synaptic input, served in the present study as the framework to define regions within the hippocampal formation. We were particularly interested in the CA2, CA3 and CA4 regions within the hippocampus proper, as well as in the ProS, PaS and TrS within the subicular complex, since their existence as independent entities has been subject of longstanding controversy:

- CA3 has often been merged with CA2, following the scheme proposed by Stephan (1975). However, whereas in rodents and non-human primates the proximal apical dendrites of CA3 display large complex spines, those of CA2 do not have such differentiations (Ramón y Cajal 1911; Lorente de Nó 1934). In the mouse brain, a number of genes, such as those coding for the Purkinje cell protein 4, the Regulator of G-protein signaling 14, and the striatum-enriched protein-tyrosine phosphatase, are selectively expressed in pyramidal neurons of the CA2 region (Cembrowski et al. 2016). Furthermore, in the macaque brain CA3 is targeted by a considerably higher proportion of amygdalohippocampal axons than is CA2 (Wang and Barbas 2018), whereas in the rodent brain the opposite holds true for axonal input from the supramammillary nucleus and the paraventricular nucleus of the hypothalamus (Cui et al. 2013; Zhang and Hernandez 2013). Although the lucidum layer is not easily detectable in cell body stained sections, it can be clearly identified in Timm stained sections due to the high zinc concentrations in mossy fiber synaptic vesicles (Danscher 1981; Becker et al. 2005). Furthermore, the lucidum layer is particularly prominent due to its extremely high kainate and α_1_ receptor densities. Additionally, CA3 and CA2 also differed in their densities of GABA_A_, GABA_B_, M_1_, M_3_ and 5-HT_1A_ receptors, as well as of GABA_A_/BZ binding sites, which were lower in the former area. Additionally, 5-HT_2_ receptor densities were lower in CA2 than in CA3. Taken together, these data would argue against the merging of CA2 and CA3 into a single region.
- Amaral and Inausti (1990) described CA3 and CA4 as a single region, although they are not only identifiable based on the presence/absence of specific cytoarchitectonic layers, but also differed in their connectivity patterns and densities of multiple receptor types. Thus, whereas CA4 only has a pyramidal layer, CA3 is composed of the oriens, pyramidal, lucidum, radiatum and lacunosum-molecular layers. The CA4 region and the multiform layer of FD are heavily innervated by the locus coeruleus, whereas CA3 displays only a moderately dense plexus of dopamine-ß-hydroxylase fibres (Swanson and Hartman 1975). We found the lucidum layer to be also clearly distinguishable by its conspicuously high kainate and α_1_ receptor densities, which is in accordance with previously described observations in both human and rodent brains (Tremblay et al. 1985; Represa et al. 1987; Zilles et al. 2002; Palomero-Gallagher et al. 2003; Zeineh et al. 2017). The border between these two regions can also be identified based on NMDA, M_1_ and α_2_ receptor densities, which were lower in the pyramidal layer of CA3 than in CA4.
- In some studies the CA4 region was merged with the multiform layer of FD to build a single region, namely the hilus (Amaral 1978; West and Gundersen 1990; Frahm and Zilles 1994). However, CA4 and the multiform layer differ in their connectivity patterns, since the raphe nuclei project heavily to the multiform layer of FD, but only moderately to the CA4 region (Moore and Halaris 1975). Our results provide further support for the concept of CA4 as being a separate region, since it contained higher AMPA, NMDA, GABA_B_, M_1_ and M_3_ receptor densities and higher GABA_A_/BZ binding site concentrations, but lower α_1_ receptor densities than did the multiform layer of FD.
- The existence of ProS has been a subject of particular debate. Whereas some authors define it as a distinct subfield of the subicular complex (Rose 1927; Rosene and Van Hoesen 1987; Ding 2013), others (von Economo and Koskinas 1925; Stephan 1975; Braak 1980; Insausti and Amaral 2012) include the superficial layers of ProS in the CA1 region, and the deeper ones in the Sub, since they consider ProS to be a mere transition zone resulting from the overlap of elements from the hippocampus proper and the subicular complex. However, the gradual disappearance of CA-like pyramids and concomitant appearance of subicular-like pyramids in the pyramidal layer of ProS is not the only criterion by which the existence and extent of this region can be defined. Rather, the borders of ProS can also be identified by abrupt changes in lamination pattern, in the expression levels of diverse molecular and chemical markers, as well as by specific connectivity patterns. Cytoarchitectonically, the border between ProS and CA1 can be characterized by the abrupt disappearance of the radiatum layer in the former region. SMI-32 immunoreactivity in ProS is lower than in Sub, but higher than in CA1, and acetylcholinesterase staining is considerably higher in ProS than in either of the two neighbouring regions (Ding 2013). Furthermore, ProS presents higher expression levels of the neurotensin and tyrosine hydroxylase genes than do CA1 or Sub (Ding 2013). Region and layer-specific expression levels of the htr2a gene also enable the delineation of ProS (Ding 2013), and were comparable to those we found for the 5-HT_2_ receptor as labelled with [^3^H]ketanserin, an antagonist which primarily targets the 5-HT_2A_ subtype (Yadav et al. 2011). Our results concerning the remaining examined receptor types would also argue against the definition of ProS as a transition region, since it differed considerably in its receptor expression levels from both adjoining regions. Finally, the CA1, ProS and Sub regions also differ in their amygdalohippocampal connectivity patterns, with the highest density of terminal projections found in ProS and the lowest in Sub (Ding 2013; Wang and Barbas 2018). Whereas ProS projects to the perirhinal cortex, CA1 and Sub do so to the entorhinal cortex (Blatt and Rosene 1998).
- PaS and TrS were initially described as being transition regions between PreS and the entorhinal cortex, and between PreS and area BA35, respectively (Braak 1978, 1980; Braak and Braak 1993). However, PreS, PaS and TrS show different patterns of Alzheimer-related extracellular amyloid and intraneuronal neurofibrillary changes (Kalus et al. 1989), and we here found marked differences in receptor expression levels in PaS and TrS with respect to PreS. Therefore, we here classify them as distinct architectonic entities and not mere transition regions.

The densities of some receptors presented a heterogeneous distribution along the transverse axis of the CA1 and CA3 regions, thus revealing proximodistal differences which enabled the definition of three subfields within both CA1 and CA3. These subfields were not included in the delineations used to create probabilistic maps of the hippocampus, since the borders between them are not sharp or visible in cell-body stained sections (Lorente de Nó 1934), and it is technically not possible to use the same brains for probabilistic cytoarchitectonic mapping and receptorarchitectonic studies. Whereas cytoarchitectonic probabilistic maps are based on the analysis of sections obtained from entire formalin-fixed and serially sectioned brains, quantitative in vitro receptor autoradiography, which reveals the binding sites of functional receptors located in the cell membrane, requires use of unfixed, frozen brain tissue (Palomero-Gallagher and Zilles 2018). Lorente de Nó (1934) identified three subdivisions within both CA1 and CA3 based on differences in dendritic arborisation and connectivity patterns. He also specified that the borders between subfields were not sharp and could not be identified in cell-body stained sections, and this was also reflected in the changes observed here concerning receptor densities, since they were only gradual in the pyramidal layer, which encompasses the cell bodies of pyramidal neurons, and most obvious in the radiatum and lacunosum-molecular layers, where the apical dendrites of CA-pyramids are found.

The “homeostasis” of receptors in a given area, i.e., the balance between the densities of multiple receptors in that area, and not the mere presence or absence of a single receptor type, represents the molecular basis of the functionally specific local information processing in that particular area (Zilles et al. 2002; Bucher and Goaillard 2011; Maccaferri 2011). The hippocampal formation is characterized by a heterogeneous regional and laminar cyto- and receptorarchitecture which is tightly associated with segregated input, output and intrinsic fibre systems. Furthermore, the different hippocampal regions and/or layers are differentially involved in diverse aspects of memory formation and retrieval processes (Hunsaker et al. 2008; Ji and Maren 2008; Bartsch et al. 2011; Coras et al. 2014; Ledergerber and Moser 2017; Roy et al. 2017), and display a selective vulnerability to disease (Thal et al. 2000; Braak et al. 2006; Kerchner et al. 2012; Reyes-Garcia et al. 2018), as well as differential responses to pharmacological interventions and to endogenous substances (Lynch and Bliss 1986; Sato and Aoki 1997; Sato et al. 1997; Yamada et al. 2003; Kobayashi et al. 2004; Knox et al. 2011; Trieu et al. 2015). However, the exact mechanisms by which the specific molecular structure and wiring pattern of a hippocampal region underpins its functionality remain elusive. Furthermore, most of the studies have been carried out in rodents, and comparably detailed analyses in humans are restricted to cases of patients with focal lesions, or recordings obtained during presurgical evaluation of candidates for epilepsy surgery. Furthermore, possible pathology-related changes in functional connectivity may not be excluded, and despite advances in the field of high-resolution functional magnetic resonance imaging, subregions within the hippocampus and the subicular complex remain largely undistinguishable with current in vivo human imaging methods. Interestingly, it is known that mouse models have a poor predictive power for drug efficacy in human neurodegenerative diseases (Dawson et al. 2018). Indeed, distinct and species-specific responses of a hippocampal region to a specific drug or endogenous substance are to be expected, since the receptorarchitecture of the rodent hippocampus differs considerably from that of the human hippocampus. For example, α_1_ and 5-HT_2_ receptor densities are homogeneously distributed throughout the rat hippocampus (Topic et al. 2007), but in humans they are considerably higher in DG than in CA (Fig. 6); in the rat brain 5-HT_1A_ receptor densities are higher in DG than in CA (Topic et al. 2007), whereas the opposite holds true for the human hippocampus (Fig. 6). Thus, the present comprehensive characterization of the molecular organisation of the human hippocampus proper and subicular complex provides valuable standard measures not only for future comparisons with tissue from patients to deepen our understanding of the pathogenesis of neurological and psychiatric diseases, but also for translational neuroscience strategies in the field of drug development.

Concluding, we here provide probabilistic maps in stereotaxic space of ten cyto- and receptorarchitectonically distinct regions within the human hippocampal formation which go beyond previously published ones (Amunts et al. 2005). The present maps constitute a valuable tool for future studies involving architectonically informed structural analyses of neuroimaging in vivo datasets, or aiming to understand the differential roles of each hippocampal and subicular region in learning and memory processes. Furthermore, the characterization of the regional and laminar distribution patterns of multiple neurotransmitter receptors elucidates the molecular organisation of the hippocampal complex and provides a gold standard relevant for both basic research and translational neuroscience strategies.

## Electronic supplementary material

Below is the link to the electronic supplementary material.

Supplementary file1 (DOCX 88 kb)
