# Supplementary material for: Multimodal mapping and analysis of the cyto- and receptorarchitecture of the human hippocampus
Source: Brain Struct Funct. 2020 Jan 18;225(3):881–907. doi: 10.1007/s00429-019-02022-4 (PMC7166210; doi:10.1007/s00429-019-02022-4)
Supplement: Supplementary file 1 — Supplementary file1 (DOCX 88 kb) [file 429_2019_2022_MOESM1_ESM.docx]

Supplementary material

**Supplementary Table 1:** Ranges in anatomical MNI space of regions defined within the hippocampal formation. Values are given for each of the individual brains (B01-B14) and for the continuous probability maps (p-maps) in the left and right hemispheres.

|  | FD | | | | | | CA4 | | | | | | CA3 | | | | | | CA2 | | | | | | CA1 | | | | | |
| --- | --- | --- | --- | --- | --- | --- | --- | --- | --- | --- | --- | --- | --- | --- | --- | --- | --- | --- | --- | --- | --- | --- | --- | --- | --- | --- | --- | --- | --- | --- |
|  | x | | y | | z | | x | | y | | z | | x | | y | | z | | x | | y | | z | | x | | y | | z | |
| LEFT | min | max | min | max | min | max | min | max | min | max | min | max | min | max | min | max | min | max | min | max | min | max | min | max | min | max | min | max | min | max |
| B01 | -39 | -12 | -38 | -2 | -34 | 3 | -36 | -13 | -37 | -4 | -32 | -1 | -34 | -11 | -38 | -4 | -30 | 4 | -37 | -11 | -39 | -5 | -32 | 4 | -41 | -12 | -41 | 2 | -36 | 4 |
| B02 | -35 | -12 | -38 | 1 | -40 | 2 | -34 | -16 | -36 | -1 | -38 | 2 | -34 | -9 | -37 | -2 | -37 | 4 | -36 | -8 | -38 | -2 | -38 | 3 | -38 | -8 | -39 | 5 | -44 | 4 |
| B03 | -35 | -5 | -37 | -2 | -38 | 7 | -33 | -6 | -35 | -4 | -37 | 5 | -34 | -5 | -37 | -4 | -34 | 10 | -35 | -6 | -38 | -3 | -35 | 11 | -36 | -5 | -39 | 3 | -40 | 10 |
| B04 | -37 | -6 | -37 | -3 | -31 | 7 | -36 | -11 | -36 | -5 | -28 | 7 | -36 | -6 | -36 | -4 | -26 | 8 | -37 | -7 | -37 | -5 | -27 | 8 | -40 | -5 | -40 | 2 | -38 | 8 |
| B05 | -39 | -6 | -38 | -3 | -34 | 7 | -37 | -8 | -37 | -5 | -31 | 4 | -37 | -5 | -38 | -6 | -29 | 8 | -39 | -7 | -38 | -5 | -31 | 9 | -40 | -8 | -40 | 2 | -37 | 9 |
| B07 | 9 | 40 | -36 | -1 | -34 | 9 | -36 | -9 | -37 | -4 | -32 | 1 | -36 | -8 | -40 | -5 | -28 | 5 | -37 | -9 | -39 | -4 | -30 | 4 | -37 | -10 | -44 | 1 | -36 | 4 |
| B08 | -38 | -8 | -38 | -7 | -31 | 4 | -35 | -14 | -36 | -9 | -29 | 3 | -33 | -8 | -37 | -8 | -29 | 5 | -37 | -10 | -38 | -8 | -31 | 7 | -41 | -9 | -42 | -2 | -34 | 7 |
| B09 | -37 | -6 | -39 | -4 | -32 | 5 | -35 | -9 | -37 | -5 | -30 | 4 | -35 | -5 | -38 | -5 | -29 | 7 | -37 | -5 | -40 | -4 | -30 | 7 | -38 | -6 | -42 | -2 | -35 | 8 |
| B13 | -35 | -12 | -37 | -2 | -30 | 5 | -34 | -17 | -36 | -5 | -29 | 3 | -35 | -9 | -38 | -4 | -26 | 6 | -37 | -12 | -39 | -3 | -28 | 7 | -37 | -14 | -43 | 3 | -36 | 6 |
| B14 | -37 | -4 | -39 | -3 | -31 | 7 | -36 | -7 | -38 | -5 | -30 | 4 | -37 | -4 | -39 | -4 | -27 | 9 | -37 | -6 | -39 | -3 | -26 | 11 | -38 | -8 | -40 | 0 | -34 | 9 |
| p-map | -37 | -8 | -38 | -3 | -33 | 4 | -36 | -17 | -36 | -4 | -31 | 3 | -35 | -6 | -37 | -6 | -30 | 7 | -38 | -7 | -38 | -3 | -31 | 9 | -40 | -8 | -41 | 2 | -36 | 7 |
| RIGHT |  |  |  |  |  |  |  |  |  |  |  |  |  |  |  |  |  |  |  |  |  |  |  |  |  |  |  |  |  |  |
| B01 | 8 | 39 | -38 | 0 | -34 | 3 | 12 | 37 | -36 | -2 | -32 | 3 | 8 | 36 | -36 | -2 | -31 | 4 | 8 | 38 | -36 | -3 | -31 | 5 | 8 | 41 | -40 | 5 | -37 | 4 |
| B02 | 8 | 34 | -36 | 3 | -41 | 3 | 10 | 33 | -35 | 0 | -39 | 3 | 7 | 34 | -36 | -1 | -38 | 6 | 8 | 35 | -36 | 0 | -40 | 6 | 8 | 38 | -36 | 8 | -43 | 6 |
| B03 | 4 | 37 | -37 | -1 | -35 | 6 | 5 | 36 | -35 | -4 | -33 | 5 | 4 | 37 | -36 | -3 | -32 | 9 | 6 | 38 | -36 | -3 | -33 | 10 | 5 | 39 | -38 | 4 | -39 | 8 |
| B04 | 8 | 41 | -36 | -1 | -35 | 7 | 12 | 39 | -35 | -4 | -32 | 6 | 8 | 38 | -35 | -3 | -29 | 7 | 10 | 41 | -36 | -3 | -29 | 7 | 8 | 43 | -37 | 3 | -36 | 7 |
| B05 | 6 | 41 | -38 | -3 | -32 | 3 | 6 | 37 | -37 | -5 | -31 | 1 | 5 | 37 | -37 | -5 | -29 | 6 | 6 | 38 | -38 | -4 | -29 | 10 | 6 | 42 | -40 | 1 | -35 | 10 |
| B07 | -36 | -9 | -37 | -3 | -34 | 1 | 12 | 39 | -31 | -3 | -32 | 2 | 8 | 39 | -37 | -4 | -29 | 10 | 12 | 41 | -35 | -2 | -29 | 7 | 13 | 41 | -36 | 4 | -38 | 6 |
| B08 | 5 | 38 | -37 | -5 | -32 | 4 | 7 | 35 | -35 | -7 | -30 | 2 | 5 | 33 | -38 | -7 | -30 | 4 | 6 | 35 | -39 | -4 | -32 | 5 | 5 | 42 | -41 | 1 | -35 | 5 |
| B09 | 6 | 39 | -37 | -2 | -32 | 5 | 8 | 38 | -36 | -4 | -31 | 3 | 2 | 38 | -39 | -5 | -29 | 7 | 5 | 40 | -39 | -3 | -30 | 7 | 7 | 40 | -40 | 0 | -36 | 7 |
| B13 | 6 | 46 | -37 | -2 | -34 | 3 | 15 | 43 | -35 | -6 | -31 | 2 | 7 | 42 | -37 | -2 | -29 | 5 | 7 | 48 | -37 | -2 | -30 | 5 | 7 | 48 | -47 | 2 | -37 | 4 |
| B14 | 8 | 41 | -37 | -2 | -31 | 6 | 10 | 37 | -36 | -4 | -29 | 3 | 6 | 36 | -36 | -3 | -27 | 7 | 8 | 39 | -37 | -2 | -28 | 8 | 9 | 42 | -39 | 0 | -33 | 8 |
| p-map | 8 | 40 | -36 | -2 | -33 | 4 | 17 | 37 | -36 | -4 | -32 | 2 | 8 | 36 | -36 | -4 | -28 | 7 | 8 | 40 | -37 | -3 | -29 | 7 | 9 | 42 | -38 | 4 | -37 | 5 |

**Supplementary Table 2.** Ranges in anatomical MNI space of regions defined within the subicular complex. Values are given for each of the individual brains (B01-B14) and for the continuous probability maps (p-maps) in the left and right hemispheres.

|  | Prosubiculum | | | | | | Subiculum | | | | | | Presubiculum | | | | | | Parasubiculum | | | | | | Transsubiculum | | | | | |
| --- | --- | --- | --- | --- | --- | --- | --- | --- | --- | --- | --- | --- | --- | --- | --- | --- | --- | --- | --- | --- | --- | --- | --- | --- | --- | --- | --- | --- | --- | --- |
|  | x | | y | | z | | x | | y | | z | | x | | y | | z | | x | | y | | z | | x | | y | | z | |
| LEFT | min | max | min | max | min | max | min | max | min | max | min | max | min | max | min | max | min | max | min | max | min | max | min | max | min | max | min | max | min | max |
| B01 | -35 | -11 | -40 | 3 | -38 | 6 | -35 | -10 | -41 | 3 | -44 | 6 | -30 | -9 | -38 | 1 | -43 | -1 | -27 | -12 | -17 | 0 | -42 | -25 | -25 | -16 | -41 | -15 | -32 | -6 |
| B02 | -35 | -13 | -37 | 6 | -44 | 4 | -32 | -11 | -37 | 9 | -50 | 3 | -28 | -14 | -33 | 7 | -46 | -12 | -26 | -10 | -15 | 7 | -46 | -26 | -22 | -16 | -32 | -14 | -35 | -16 |
| B03 | -32 | -7 | -40 | 4 | -41 | 8 | -30 | -7 | -41 | 5 | -41 | 7 | -25 | 31 | -38 | 2 | -40 | 3 | -25 | -11 | -20 | 1 | -40 | -24 | -20 | -9 | -39 | -19 | -29 | 0 |
| B04 | -32 | -7 | -41 | 2 | -40 | 8 | -31 | -6 | -42 | 4 | -40 | 7 | -25 | -7 | -41 | 2 | -35 | 2 | -23 | -8 | -23 | 3 | -34 | -21 | -23 | -11 | -45 | -22 | -23 | 0 |
| B05 | -33 | -9 | -40 | 3 | -41 | 5 | -29 | -8 | -41 | 3 | -41 | 5 | -23 | -6 | -39 | 1 | -38 | 1 | -21 | -9 | -24 | 1 | -38 | -24 | -18 | -10 | -42 | -24 | -26 | -7 |
| B07 | -34 | -11 | -42 | 2 | -38 | -2 | -29 | -9 | -44 | 2 | -39 | 5 | -23 | -8 | -43 | 0 | -37 | 4 | -21 | -11 | -30 | -2 | -35 | -18 | -19 | -9 | -41 | -23 | -25 | -5 |
| B08 | -34 | -12 | -43 | 0 | -35 | 7 | -31 | -10 | -43 | 1 | -36 | 6 | -27 | -8 | -39 | -1 | -36 | 2 | -23 | -9 | -25 | -1 | -35 | -24 | -20 | -9 | -43 | -22 | -24 | -2 |
| B09 | -32 | -9 | -42 | -2 | -37 | 7 | -29 | -8 | -42 | -1 | -38 | 5 | -23 | -7 | -39 | -3 | -35 | 0 | -21 | -10 | -24 | -2 | -34 | -23 | -18 | -9 | -43 | -23 | -27 | -3 |
| B13 | -30 | -15 | -43 | 2 | -34 | 5 | -28 | -12 | -43 | 1 | -34 | 4 | -24 | -9 | -37 | -3 | -31 | -5 | -23 | -11 | -27 | -4 | -30 | -15 | -24 | -11 | -39 | -25 | -21 | -5 |
| B14 | -33 | -10 | -39 | 1 | -37 | 4 | -29 | -9 | -39 | 1 | -38 | 5 | -23 | -5 | -38 | 0 | -35 | 5 | -21 | -10 | -23 | 0 | -34 | -21 | -17 | -7 | -41 | -21 | -25 | 0 |
| p-map | -33 | -11 | -41 | 2 | -39 | 7 | -30 | -10 | -41 | 3 | -39 | 5 | -25 | -7 | -38 | 0 | -37 | 0 | -24 | -10 | -25 | 0 | -40 | -22 | -22 | -9 | -42 | -17 | -30 | -3 |
| RIGHT |  |  |  |  |  |  |  |  |  |  |  |  |  |  |  |  |  |  |  |  |  |  |  |  |  |  |  |  |  |  |
| B01 | 12 | 34 | -38 | 5 | -39 | 1 | 7 | 32 | -39 | 5 | -42 | 0 | 7 | 29 | -34 | 4 | -39 | -5 | 15 | 26 | -14 | 3 | -39 | -21 | 15 | 26 | -39 | -11 | -30 | -8 |
| B02 | 10 | 36 | -35 | 8 | -49 | 5 | 10 | 35 | -34 | 11 | -52 | 5 | 11 | 33 | -30 | 10 | -50 | -14 | 15 | 30 | -12 | 9 | -47 | -25 | 12 | 29 | -32 | -12 | -36 | -10 |
| B03 | 6 | 34 | -38 | 4 | -38 | 8 | 7 | 32 | -38 | 5 | -38 | 7 | 5 | 29 | -37 | 2 | -39 | -2 | 12 | 28 | -15 | 2 | -38 | -23 | 5 | 23 | -37 | -14 | -28 | -3 |
| B04 | 10 | 36 | -37 | 4 | -39 | 6 | 8 | 34 | -37 | 5 | -40 | 5 | 6 | 30 | -37 | 0 | -35 | 0 | 15 | 27 | -18 | 1 | -33 | -21 | 6 | 24 | -38 | -17 | -26 | -1 |
| B05 | 7 | 37 | -39 | 2 | -38 | 9 | 7 | 33 | -39 | 3 | -39 | 7 | 8 | 28 | -38 | -1 | -37 | 3 | 11 | 24 | -23 | 1 | -36 | -24 | 10 | 23 | -42 | -23 | -27 | -3 |
| B07 | 14 | 38 | -34 | 4 | -39 | 3 | 9 | 32 | -37 | 5 | -40 | 5 | 9 | 28 | -34 | 2 | -38 | 1 | 12 | 25 | -26 | -1 | -36 | -16 | 8 | 22 | -32 | -18 | -28 | -7 |
| B08 | 8 | 37 | -44 | 2 | -37 | 7 | 6 | 34 | -44 | 5 | -37 | 6 | 5 | 29 | -39 | 1 | -36 | -2 | 11 | 25 | -19 | 1 | -35 | -22 | 8 | 22 | -41 | -17 | -29 | -5 |
| B09 | 7 | 34 | -41 | 1 | -38 | 6 | 6 | 31 | -41 | 1 | -38 | 4 | 5 | 26 | -37 | -1 | -34 | -4 | 12 | 23 | -23 | -1 | -33 | -23 | 10 | 21 | -39 | -22 | -25 | -7 |
| B13 | 7 | 36 | -41 | 2 | -37 | 4 | 7 | 33 | -40 | 1 | -37 | 4 | 6 | 31 | -37 | 0 | -33 | -3 | 14 | 30 | -25 | -1 | -31 | -16 | 11 | 21 | -39 | -23 | -20 | -6 |
| B14 | 10 | 37 | -37 | 2 | -35 | 4 | 8 | 33 | -39 | 2 | -35 | 8 | 12 | 27 | -33 | 0 | -34 | 1 | 12 | 24 | -20 | 0 | -33 | -22 | 11 | 21 | -37 | -18 | -25 | -2 |
| p-map | 11 | 36 | -40 | 3 | -38 | 5 | 7 | 32 | -40 | 5 | -39 | 4 | 6 | 28 | -36 | 0 | -37 | -4 | 12 | 26 | -22 | 0 | -38 | -22 | 9 | 23 | -40 | -15 | -28 | -5 |
